# Inositol hexakisphosphate kinase 1 is implicated in the insulin response to protein ingestion in older adults

**DOI:** 10.1038/s41598-026-35711-2

**Published:** 2026-02-18

**Authors:** Richie D. Barclay, Diana E. Motei, Oana Ancu, Christopher J. Tyler, Neale A. Tillin, Volker Behrends, Nicholas A. Burd, Nicholas M. Hurren, Richard W.A. Mackenzie

**Affiliations:** 1 School of Life and Health Sciences, University of Roehampton, London, UK; 2 Division of Nutritional Sciences, University of Illinois Urbana-Champaign, Urbana, IL USA; 3 Department of Health and Kinesiology, University of Illinois Urbana-Champaign, Urbana, IL USA; 4 School of Biomedical Science, University of West London, London, UK; 5 Centre for Health & Life Sciences, Research Institute for Health & Wellbeing, Coventry University, Coventry, UK; 6 Institute of Cardio-Metabolic Medicine, Coventry University, University Hospital Coventry & Warwick NHS Trust, Coventry, CV1 5FB UK; 7 London Institute for Human Performance and Longevity, 1 Blythe Road, London, W14 0HG UK

**Keywords:** IP6K1, Metabolism, Resistance exercise, Amino acids, Ageing, Insulin resistance, Ageing, Metabolism, Molecular biology, Endocrinology, Endocrine system and metabolic diseases

## Abstract

Age-related muscle mass is driven by a reduction in insulin sensitivity partly mediated by reduced amino acid and anabolic signalling kinetics. Insulin activates Akt-mTORC1 signalling in skeletal muscle, with inositol hexakisphosphate kinase 1 (IP6K1) shown to inhibit this signalling pathway in pre-diabetic humans. We aimed to compare muscle and plasma IP6K1 in young vs older adults and the possible role of IP6K1 in the anabolic response to protein and protein plus resistance exercise (RE). Nine young (24.9 ± 0.4 years) and nine older (66.2 ± 0.5 years), moderately active adults received primed continuous infusions of L-[*ring*-^2^H_5_]phenylalanine in basal and postprandial state. Blood and muscle biopsy samples were collected prior to and following ingestion of 25 g whey protein with or without knee extension exercise to examine skeletal muscle protein signalling and whole-body phenylalanine kinetics. Young adults had greater plasma IP6K1 at all time points. Older adults had reduced muscle IP6K1 at 120 min post-exercise. Muscle IP6K1 decreased 240 min postprandially in young adults compared with basal and there was no effect of exercise in either group. Older adults presented with reduced plasma and muscle IP6K1 in both postprandially and post-RE states, as well as reduced phenylalanine rate of disappearance for the same comparisons. IP6K1 may be involved in the reduction in amino acid metabolism_,_ and the insulin-mediated response to protein and RE.

**Supplementary Information:**

The online version contains supplementary material available at 10.1038/s41598-026-35711-2.

## Introduction

Type 2 diabetes (T2D) is largely characterised by ß-cell dysfunction, and both hepatic and peripheral insulin resistance^1^. The intracellular mechanisms that govern insulin-stimulated glucose transport are poorly understood. Various research models suggest that inositol hexakisphosphate kinase 1 (IP6K1) plays a negative role in this key metabolic process^1–4^. Elsewhere, insulin and insulin-like growth factor 1 in like growth factor 1 (IGF-1) are now known to activate similar intracellular pathways via synergistic activation at the respective IGF receptor (IGFR) and insulin receptor substrate 1 (IRS-1) receptors^5,6^. IP6K1 is a six carbon cyclitol kinase that binds to intracellular phosphatidic acid (PA) allowing for its localisation to the cell’s nucleus^4^, before producing a pyrophosphate group at the 5th position of IP6, generating IP_7_. IP_7_ is then available to bind the pleckstrin homology (PH) domain of Akt preventing its translocation to the cell membrane and blunting subsequent phosphorylation by phosphoinositide-dependent kinase 1 (PDK1) at Akt^308^^3^. This reduction in p-Akt^308^ reduces downstream signalling, bringing about a reduction in whole-body nutrient disposal^2,7^. Evidence suggests that plasma, not muscle, IP6K1 is positively correlated with HOMA_IR_ in hyperinsulinaemic humans^2^, which suggests plasma IP6K1 may be implicated in nutrient disposal. In middle aged insulin-resistant obese humans, lean meat ingestion increased IP6K1 muscle content^7^ while in middle aged pre-diabetic humans’ basal muscle IP6K1 was elevated, a finding that was coupled with a reduction in Akt^308^ phosphorylation^2^. Furthermore, when people with prediabetes performed high-intensity intermittent exercise, muscle IP6K1 content was reduced, and both muscle pAktThr308 and whole-body insulin sensitivity were increased^2^. These findings suggest that IP6K1 is responsive to exercise^2^, which when combined with our previous results^7^ suggests that reductions in IP6K1 are associated with improvements of whole-body nutrient (both glucose and amino acids) uptake. Yet, this is a question that needs further research as the mechanisms that govern nutrition uptake, insulin sensitivity, and anabolic resistance, are still poorly understood- and anabolic- senstivity are still poorly understood^8^.

The primary aim of this study was to characterise and compare plasma and muscle IP6K1 in young vs older insulin-resistant adults, and to assess how IP6K1 may affect neighbouring protein signalling, associated with MPS. It was hypothesised that insulin-resistant older adults would present increased plasma and muscle IP6K1 in all conditions versus young adults. It was also hypothesised that an acute bout of RE would decrease muscle and plasma IP6K1 which would be coupled with an increase in molecular protein signalling. L-type amino acid transporter 1 (LAT1) is a key part of the anabolic signaling pathway. Indeed, amino acid transporters provide a link between plasma amino acid availability, mTORC1-related signalling, and the muscle protein synthetic response. Prior work has shown differences in skeletal muscle LAT1 responses to resistance exercise and amino acid ingestion between young and older men^9^. An exploratory aim was to assess if age-related differences in postprandial whole-body phenylalanine kinetics existed with and without prior resistance exercise, in the context of LAT1.

## Methodology

### Participants and ethical approval

Nine young (24.9 ± 0.4 years) and nine older (66.2 ± 0.5 years) men volunteered to take part in this study. Sample size was determined by conducting a statistical power analysis. Participants were deemed moderately active (14–23 units) by the Godin Leisure Time Questionnaire^10^. Young and old participant characteristics are shown in Table 1. Exclusion criteria are described in a previous study by the same group^2^. Each participant gave written and verbal consent following full explanation of the study, its risks and its aims. The study was approved locally by the University of Roehampton Ethics Committee (UoR 16/181) and complied to guidelines of the Declaration of Helsinki for use of human volunteers.

**Table 1:** Participant characteristics.

Variable	Young (n = 9)	Older (n = 9)
Age (years)	24.9 ± 3.6	66.2 ± 5.3*
Height (m)	1.77 ± 0.09	1.80 ± 0.08
Body mass (kg)	80.6 ± 14.4	87.4 ± 15.4
Body mass index (kg/m^2^)	25.7 ± 4.8	27.3 ± 2.4
Body fat (%)	12.6 ± 4.8	25.6 ± 5.9*
Lean body mass (kg)	69.9 ± 10.1	62.1 ± 7.5
Resting heart rate (bpm)	60 ± 11	54 ± 9
Systolic BP (mmHg)	129 ± 11	126 ± 13
Diastolic BP (mmHg)	77 ± 8	80 ± 6
Grip strength (kg/m^2^)	50.8 ± 8.7	39.7 ± 5.6*
One repetition maximum (kg)	71.1 ± 13.2	54.2 ± 5.9*
Activity level (Godin leisure time)	22 ± 3	21 ± 3
Fasting plasma glucose (mmol/L)	4.1 ± 1.8	5.4 ± 1.4*
HOMA_IR_	1.7 ± 0.9	3.2 ± 1.2*

Values are means ± SD. An independent samples t-test was used to determine between group differences. * *P* < 0.05 vs young.

Participants presented to the laboratory having avoided any physical activity for 48 h. Body composition was measured by air displacement plethysmography (Cosmed; BodPod) before participants completed a one-repetition maximum (1-RM) test on a commercially available knee extension resistance machine. Muscle function was measured using a 30 s chair squat (> 11 bouts classified as healthy and non-sarcopenic^11^ and a 10 m walk test (< 12.6 s to be classified as healthy and non-sarcopenic)^12^. This visit was 7–14 days before the first experimental trial day.

**Fig. 1:**
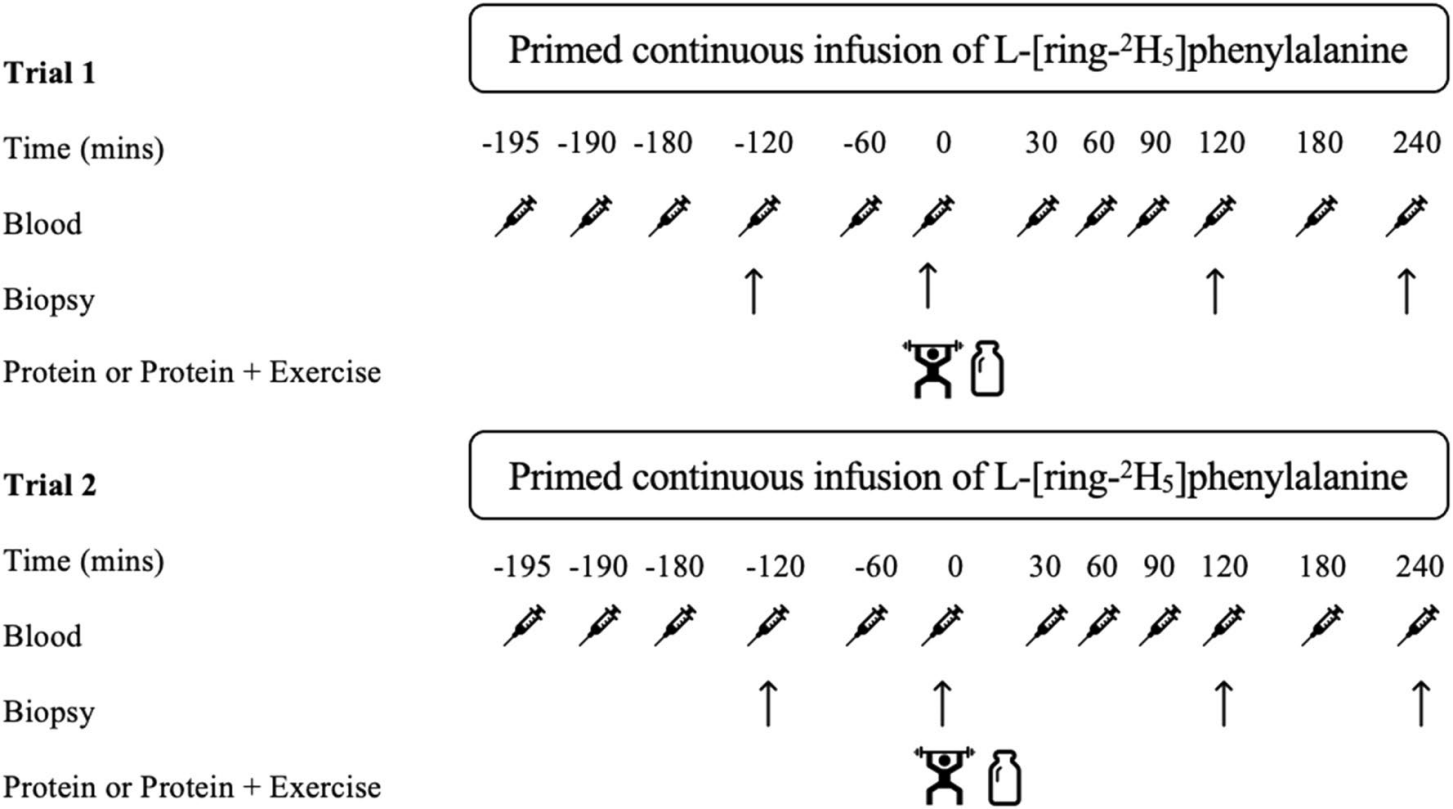
Schematic diagram of the study design which was carried out in a randomized order for the two interventions at time point 0. Intervention one was 25 g of whey protein isolate enriched with 3% L-[ring-^2^H_5_]phenylalanine tracer and intervention two was 6 sets of 8 repetitions at 60% one repetition maximum on a commercially available knee extension resistance machine followed by the same protein drink. For both trials a primed, continuous infusion of L-[ring-^2^H_5_]phenylalanine was maintained for the duration with skeletal muscle biopsies and venous blood samples drawn at set time points throughout the study. The 30 min blood sample was drawn 30 min after each participant finished the protein drink. Indicates blood sample. Indicates skeletal muscle biopsy sample. Indicates whey protein isolate drink containing 25 g protein enriched with 3% L-[ring-^2^H_5_]phenylalanine tracer. Indicates 6 sets of 8 repetitions of knee extension resistance exercise.

A repeated measures crossover design was used for this study with each experimental trial were completed in randomised order. Randomization was completed using commercially available software to determine order. One trial involved protein ingestion only, and the other involved an acute bout of 6 sets of 8 repetitions RE at 60% 1-RM immediately followed by 25 g whey protein isolate. Participants were instructed to consume a balanced meal the evening prior to experimental trials which was confirmed upon arrival to the laboratory. The meal consisted of ~ 40% carbohydrate, 30% fat and 30% protein, with meal examples provided to participants during visit 1. Compliance with meal composition the evening prior was confirmed verbally upon arrival to the laboratory for the experimental trials. Participants refrained from physical activity and alcohol for 48 h prior to each experimental trial. Participants received a primed and continuous infusion of L-[ring-^2^H_5_]phenylalanine following previously described methods^13^ (Fig. 1).

### Amino acids analysis including L-[ring-^2^H_5_]phenylalanine

Plasma amino acids, including L-[ring-^2^H_5_]phenylalanine enrichments, were determined using liquid chromatography–mass spectrometry (LC–MS). Plasma amino acid levels were determined using a modified version of a previously described method^14^. Briefly, 30 ug/ul internal standard (Glycine-2-13C, Sigma-Aldrich; #279,439) was added to human plasma samples followed by protein precipitation by ice-cold isopropanol containing 1% formic acid (v/v). Amino acids derivatisation was performed using a commercially available AccQ-Tag Ultra Derivatization Kit (Waters; #186,003,836) as per the manufacturer’s guidelines. The UPLC, a Waters Acquity stem, was operating a binary seven-minute gradient with water + 0.1% formic acid (solvent A) to acetonitrile + 0.1% formic acid across a Waters HSS T3 reverse phase UPLC column. The flow rate was 0.65 mL/min. The UPLC was coupled to a triple quad mass spectrometer (Waters Xevo TQ-S Micro), set to multiple reaction monitoring. Raw data files were converted to text filed using Proteowizard MSConvert and analysed using an in-house Matlab pipeline based on software published by Behrends et al. (2011). Values are expressed as arbitrary units (A/U). Blood glucose was measured using 20 µl into Biosen Glucose/Lactate Haemolysing Solution (Biosen C-Line analyser (EFK Diagnostics). Plasma concentration for IP6K1 (IP6K1, MyBioSource; #MBS9326680) and insulin (insulin, DRG, #DX-EIA-2935) were determined using commercially available ELISA kits.

### Western blot analysis

Homogenization of ~ 50 mg wet muscle as described previously^7^. Total protein was determined using by Bradford Assay (Bio-Rad; #5,000,201) and then 20 μg of protein diluted in 2X Laemmli buffer (Bio-Rad; #161–0737) was separated by SDS-PAGE in pre-cast gels (Bio-Rad; #4,568,023) before being transferred to polyvinyl difluoride membranes using a trans blot turbo transfer system (Bio-Rad; #1,704,150). Membranes were blocked for one hr (5% Milk-TBS) and then incubated in primary antibodies overnight at 4 °C with gentle agitation to determine phosphorylated and total protein of interest. A full list of antibodies can be found in our previous publications^7^. Membranes were developed and images were taken using a Li-Cor Fc scanner (Li-Cor, USA). After the detection of phosphorylated proteins, membranes were stripped and incubated with primary antibodies for total protein. Western blots were normalised using the total protein stain (Li-Cor; #926–11,015) method^15^. Blots were quantified using the Image Studio Pro software (Li-Cor, USA).

### Whole-body phenylalanine kinetics

The calculations of phenylalanine kinetics have been described previously^16^, using the Steele equation for the non-steady-state measurement. Briefly, whole body total phenylalanine rate of appearance (R_a_) (μmol.min^-1^.kg^-1^) was calculated where F is the infusion rate (μmol.min^-1^.kg^-1^) and E_p_ is plasma L-[ring-^2^H_5_]phenylalanine enrichment. Whole body total phenylalanine rate of disappearance (R_d_) was calculated by dividing plasma phenylalanine concentrations by the enriched plasma-free precursor pool over time. The *enriched plasma-free precursor pool* is the concentration of labelled phenylalanine circulating freely in the blood, which serves as the measurable precursor pool for protein synthesis and breakdown calculations when determining phenylalanine rate of disappearance (Rd). Metabolic clearance rate (MCR) of phenylalanine was calculated by dividing R_d_ with the average tracer enrichment values over the two time points measured. Phenylalanine was used, as common in metabolic research, because it is not synthesized in the body or oxidised in skeletal muscle. The rate of phenylalanine disappearance via conversion to tyrosine was not directly determined. Hence, Rd represents both metabolic fates to protein synthesis and hydroxylation to tyrosine.

### Statistical analysis

Values were calculated for all plasma and muscle variables and analysed by univariate (group x time x condition) repeated measures analysis of variance (ANOVA). Tukey’s *post-hoc* test was used to identify specific interactions when significance was found. Phenylalanine R_a_, R_d_, MCR, plasma and muscle IP6K1 were correlated with participant characteristics, plasma and muscle variables using Pearson’s correlation coefficient. Data are expressed as mean ± SEM (IBM SPSS Statistics; v26).

## Results

### Plasma signalling molecule and metabolite concentrations

**Fig. 2:**
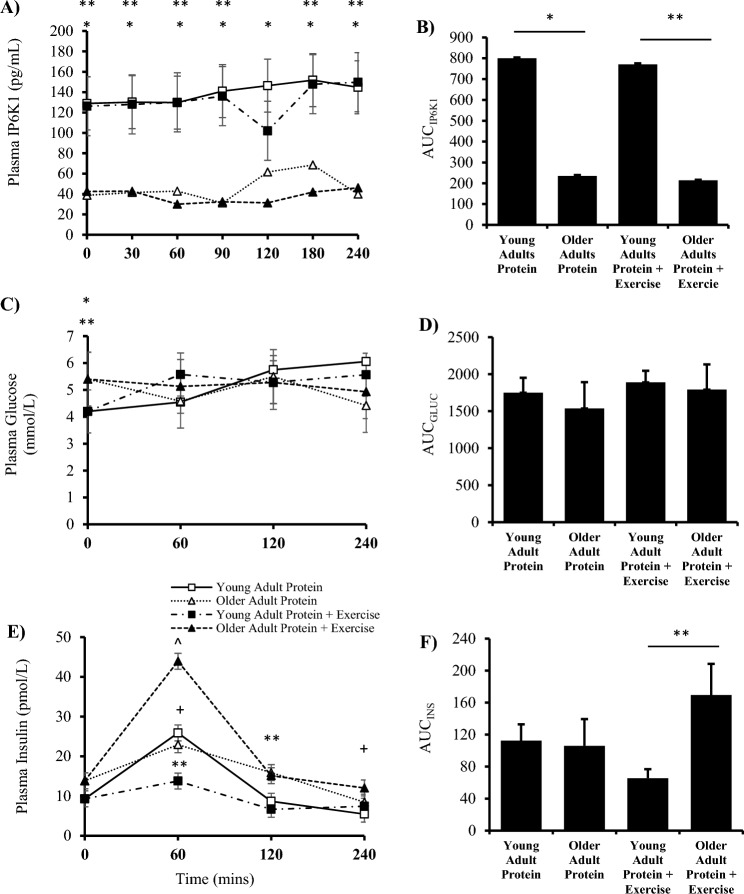
Plasma analysis for young and older adults in the post-absorptive, post-prandial and post-exercise states. Analyses of plasma IP6K1 (**A**) concentration and (**B**) area under the curve (AUC) (Protein, *P* = *0*.008; Protein + Exercise, *P* = *0*.001). Plasma glucose (**C**) Concentration and (**D**) area under the curve (Protein, *P* = *0*.899; Protein + Exercise, *P* = *0*.738). Plasma insulin E) Concentration and F) area under the curve (Protein, *P* = *0*.359; Protein + Exercise, *P* = *0*.006). (n = 9/group). * *P* < 0.05 vs older adults protein trial. ** *P* < 0.05 vs older adults exercise trial + *P* < 0.05 vs young adult basal. ^ *P* < 0.05 vs all within group. Data are mean ± SEM.

Plasma IP6K1 was unaffected by time (*P* = *0*.167) or condition (*P* = *0*.903) but was different between groups (*P* = *0*.002). Specifically, there was an increase in young versus older adults at all time points (Fig. 2A, B). Between groups, fasting plasma glucose was significantly greater in older versus young adults (*P* = *0*.001) however there was no effect of time (*P* = *0*.845), condition (*P* = *0*.589) or group (*P* = *0*.466) beyond the basal time point (Fig. 2C, D). Plasma insulin was affected by time (*P* = *0*.001), condition (*P* = *0*.004) and group (*P* = *0*.016). Specifically, plasma insulin concentrations increased at 60 min of the post-prandial period (*P* = *0*.026) and decreased at 240 min versus basal (*P* = *0*.031) within the younger cohort. Within the older adult group, plasma insulin at 60 min post-exercise increased versus all time points (*P* < 0.05) as well as versus young adults at the same time point (*P* = *0*.001, + 318%). Between groups, older adults had an increased concentration of plasma insulin versus young adults at 120 min post-exercise (*P* = *0*.043) (Fig. 2E, F). Young adult HOMA_IR_ was lower compared to older adults (*P* = *0*.031). Differences in plasma leucine and BCAAs values in young and older adults are displayed in Table 2 and 3, respectively.

**Table 2:** Plasma leucine values in young and older adults.

	Young Adult Protein (A/U)	Older Adult Protein (A/U)	Young Adult Protein + Exercise (A/U)	Older Adult Protein + Exercise (A/U)
0	0.0889 ± 0.0032	0.0909 ± 0.0032	0.0754 ± 0.0062	0.1035 ± 0.0032
30	0.3119 ± 0.0286*	0.2873 ± 0.0286*	0.2512 ± 0.0411*	0.2696 ± 0.0286*
60	0.2800 ± 0.0341*	0.3172 ± 0.0341*	0.2688 ± 0.0300*	0.3755 ± 0.0341*
90	0.2230 ± 0.0269	0.2944 ± 0.0269*	0.2204 ± 0.0258*	0.3541 ± 0.0269*
120	0.1842 ± 0.0284	0.2195 ± 0.0284*	0.1522 ± 0.0126	0.2598 ± 0.0284*
180	0.1489 ± 0.0164	0.1527 ± 0.0164*	0.1169 ± 0.0126	0.2169 ± 0.0164*
240	0.1253 ± 0.0099	0.1307 ± 0.0099	0.0972 ± 0.0101	0.1709 ± 0.0099*

*P < 0.05 vs basal within group. Significance determined by two-way ANOVA and Tukey’s *post-hoc* tests.

**Table 3:** Plasma BCAA values in young and older adults.

Time (mins)	Young Adult Protein (A/U)	Older Adult Protein (A/U)	Young Adult Protein + Exercise (A/U)	Older Adult Protein + Exercise (A/U)
0	0.3202 ± 0.0338	0.35372 ± 0.0247	0.3384 ± 0.0483	0.35242 ± 0.0378
30	0.8673 ± 0.1317*	0.82620 ± 0.0717	0.7505 ± 0.1257	0.59656 ± 0.1134
60	0.8063 ± 0.1526	0.8415 ± 0.1497*	0.8116 ± 0.1363*	0.9163 ± 0.1029*
90	0.5017 ± 0.1170	0.6808 ± 0.1467	0.7017 ± 0.1140	0.87657 ± 0.1125*
120	0.5065 ± 0.0847	0.5716 ± 0.1058	0.50561 ± 0.1053	0.7810 ± 0.0512*
180	0.3083 ± 0.0856	0.46985 ± 0.0496	0.4191 ± 0.0821	0.53631 ± 0.0880
240	0.3507 ± 0.0691	0.32176 ± 0.0753	0.3854 ± 0.0568	0.41879 ± 0.0685

*P < 0.05 vs basal within group. ** *P* < 0.05 vs older adults. Significance determined by two-way ANOVA and Tukey’s *post-hoc* tests.

### Plasma phenylalanine kinetics

**Fig. 3:**
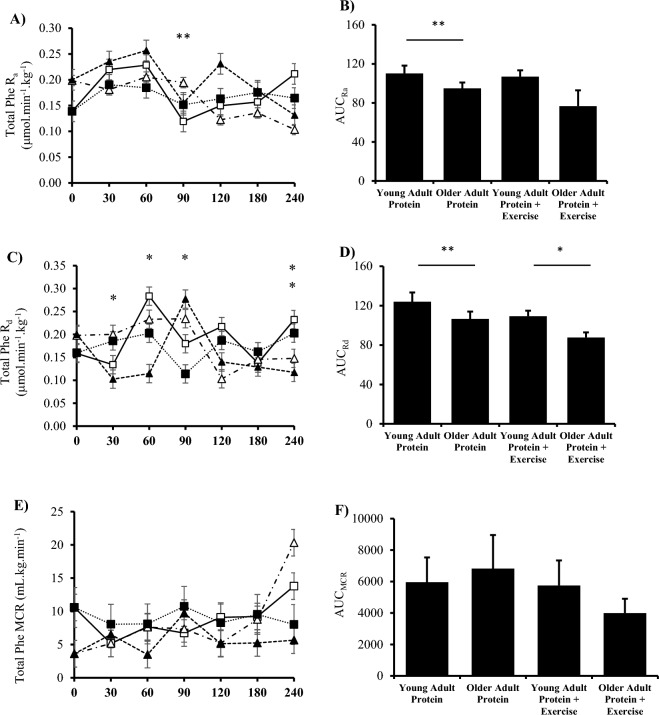

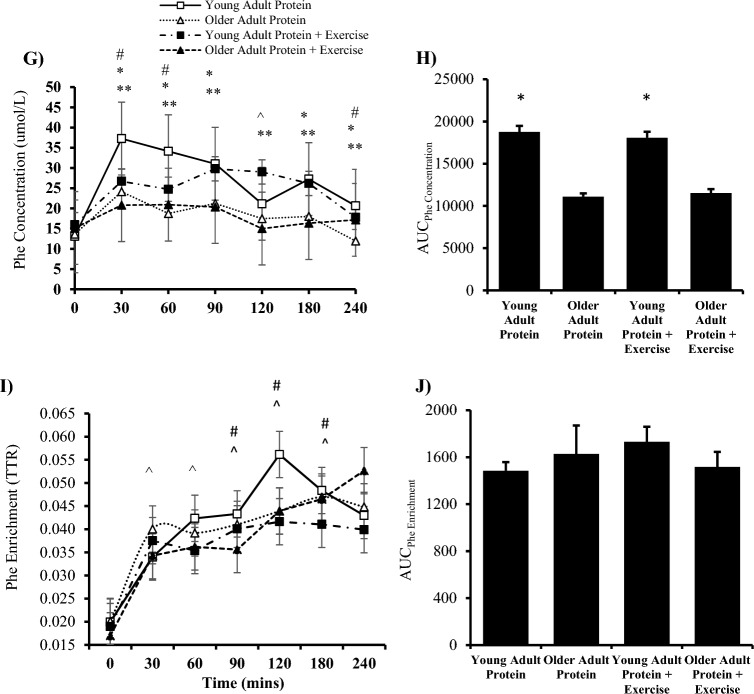
Plasma phenylalanine kinetics in the post-absorptive, post-prandial and post-exercise states. Total rate of appearance (R_a_) in (**A**) and (**B**) area under the curve (AUC) (Protein, *P* = *0*.013; Protein + Exercise, *P* = *0*.111). Total rate of disappearance (R_d_) in (**C**) and (**D**) area under the curve (Protein, *P* = *0*.22; Protein + Exercise, *P* = *0*.021). Total metabolic clearance rate (MCR) in (**E**) and (**F**) area under the curve (Protein, *P* = *0*.609; Protein + Exercise, *P* = *0*.21-). (n = 9/group). Plasma phenylalanine enrichment in (** G**) and are under the curve in (**H**) (Protein, *P* = *0*.482; Protein + Exercise, P = 0.518). Plasma phenylalanine concentration in (**I**) and area under the curve in (**J**) (Protein, *P* = *0*.021; Protein + Exercise, *P* = *0*.033 * *P* < 0.05 vs older adult protein + exercise trial. ** *P* < 0.05 vs older adults protein trial. # P < 0.05 vs basal within young adults. ^ P < 0.05 vs basal within older adults. Data are expressed as mean ± SEM.

Total phenylalanine R_a_ was affected by group (*P* = 0.038) but unaffected by condition (*P* = 0.598). Between groups, there was a reduction in post-prandial total R_a_ at 90 min (*P* = 0.029) and an increase at 240 min (*P* = 0.005) in young versus older adults (Fig. 3A; 3B). Total R_d_ was affected by time (*P* = 0.005), condition (*P* = 0.014) and group (*P* = 0.001). Between groups, there was a greater post-prandial total R_d_ in young versus older adults at 240 min (*P* = 0.012). In the exercise trial, young adults had a greater total R_d_ at 30 (*P* = 0.035), 60 (*P* = 0.031) and 240 min (*P* = 0.016) versus older adults. Moreover, at 90 min (*P* = 0.051) young adults showed a decreased total R_d_ compared to older adults (Fig. 3C; 3D). There were no significant differences for MCR within or between groups (*P* > 0.05) (Fig. 3E,F).

Plasma phenylalanine concentration presented a main time (*P* = 0.05) and group effect (*P* = 0.002) (Fig. 3G,H). In response to 25 g whey protein isolate, young adults had a significantly greater concentration of phenylalanine at 30 (*P* = 0.006), 60 (P = 0.026), and 240 min (*P* = 0.044) versus basal. The addition of exercise increased phenylalanine at 60 (*P* = 0.050) versus 240 min in young adults. Finally, there was an increase in plasma phenylalanine concentration at 30 min versus the protein + RE trial (*P* = 0.002). In older adults, protein ingestion alone significantly increased plasma phenylalanine concentration 30 (*P* = 0.033) and 120 (*P* = 0.041) versus 240 min. The addition of exercise increased the concentration at 90 min versus basal (*P* = 0.037). All time points between groups were significantly different (*P* < 0.05) with the exception to the protein trial 120 min time point (*P* = 0.056).

There was a significant effect of time (*P* = 0.008) but no effect of group (*P* = 0.873) on plasma phenylalanine enrichment (tracer:tracee; TTR). In young adults, protein ingestion increased TTR 120 min post-ingestion (*P* = 0.040). The addition of exercise in the sample group increased TTR at 90 (*P* = 0.015), 120 (*P* = 0.046) and 180 min (*P* = 0.046). The addition of exercise significantly increased TTR at 30 (P = 0.042), 60 (*P* = 0.022), 90 (*P* = 0.007) and 120 min (*P* = 0.007) versus basal. There was also a trend at of increased TTR at 240 min (*P* = 0.067) (Fig. 3I, J).

### Skeletal muscle signalling

**Fig. 4:**
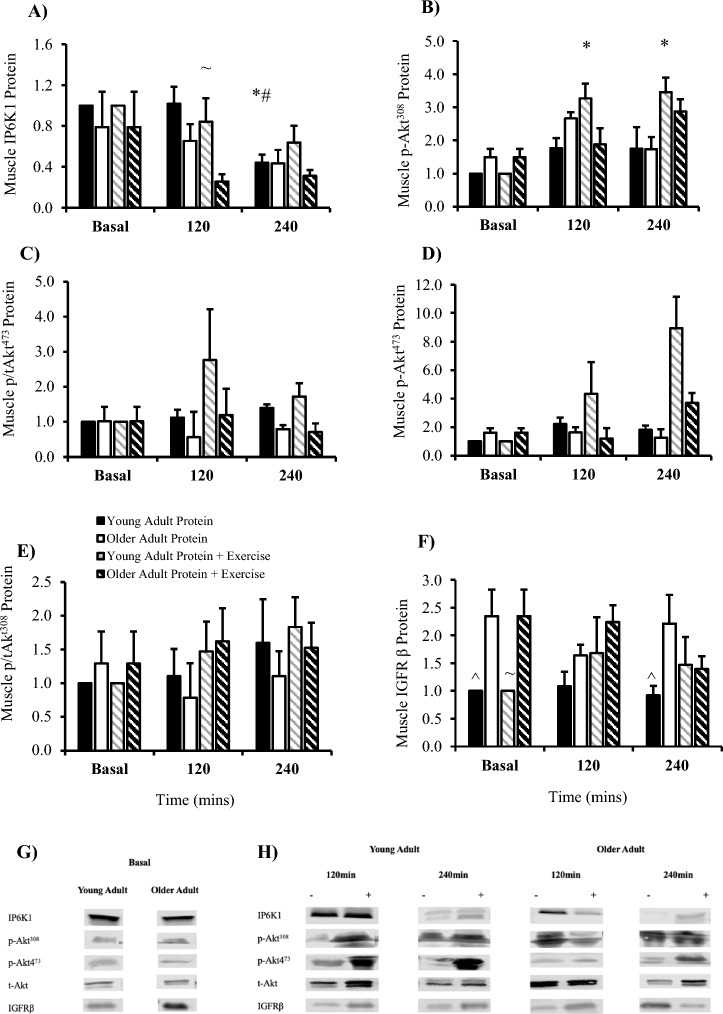
Skeletal muscle signalling of the IP6K1-IGFR-Akt pathway in young and older adults expressed as fold change from young basal. (**A**) Skeletal muscle signalling of IP6K1. (**B**) Skeletal muscle signalling of p-Akt^308^ and (**C**) Phosphorylated/total Akt^308^. (**D**) Skeletal muscle signalling of p-Akt^473^ and (**E**) Phosphorylated/total Akt^473^. Cropped blot images for proteins of interest at (**G**) basal (**H**) post-prandial (-) and post-exercise ( +). (n = 9/group). * *P* < 0.05 vs basal within group. # *P* < 0.05 vs 120 min. ^ *P* < 0.05 vs older adult protein trial. ~ *P* < 0.05 vs older adult protein + exercise trial. Data are mean ± SEM.

Muscle IP6K1 was affected by time (*P* = 0.027), condition (*P* = 0.003) and group (*P* = 0.039). Within group, post-prandial IP6K1 was lower in young adults at 240 min versus basal (*P* = 0.030) and 120 min (*P* = 0.002) (Fig. 4A). Between groups, older adults had significantly less IP6K1 at 120 min post-exercise versus young adults (*P* = 0.035). Phosphorylated Akt^308^ was affected by time (*P* = 0.015) and condition (*P* = 0.029) but not group (*P* = 0.661). Within the young adult group, post-exercise p-Akt^308^ was increased at 120 (*P* = 0.001) and 240 min (*P* = 0.011) versus basal (Fig. 4B). There was no effect of time (*P* = 0.429), condition (*P* = 0.841) or group (*P* = 0.372) on phosphorylated/total expressed as a ratio (Fig. 4C; p/tAkt^473^). Phosphorylated Akt^473^ was unaffected by time (*P* = 0.255), condition (*P* = 0.257) and group (*P* = 0.07) (Fig. 4D). There was no time (*P* = 0.409), condition (*P* = 0.696) or group (*P* = 0.565) effect on phosphorylated/total expressed as a ratio (Fig. 4E; p/tAkt^308^). Skeletal muscle IGFRβ was unaffected by time (*P* = 0.876) and condition (*P* = 0.83) but was affected by group (*P* = 0.016). Basal skeletal muscle IGFRβ (Fig. 4F) was increased in insulin-resistant older versus young adults (*P* = 0.022).

**Fig. 5:**
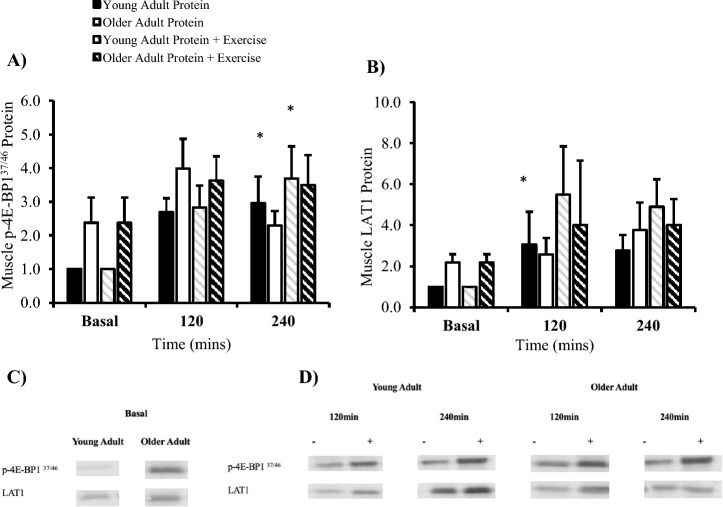
Skeletal muscle signalling of (**A**) p-4E-BP1^37/46^ and (**B**) LAT1 in young and older adults expressed as fold change from young basal. Cropped blot images for proteins of interest in (**C**) young adults and (**D**) older adults (n = 9/group). * *P* < 0.05 vs basal within group. # *P* < 0.05 vs older adults. Data are mean ± SEM.

Phosphorylated 4E-BP1^37/46^ was affected by time (*P* = 0.007) but not condition (*P* = 0.407) or group (*P* = 0.100). Within the young adult group, p-4E-BP1^37/46^ increased at 240 min post prandial (*P* = 0.023, + 196%) (Fig. 5A). In the same sample, the addition of exercise increased p-4E-BP1^37/46^ at 240 min versus basal (*P* = 0.018, + 269%). Muscle LAT1 was affected by time (*P* = 0.431) but not group (*P* = 0.058) or condition (*P* = 0.830). Within the young adult group, post-exercise LAT1 increased after 120 min (*P* = 0.027, + 206%) versus basal (Fig. 5B).

### Correlation data

**Fig. 6:**
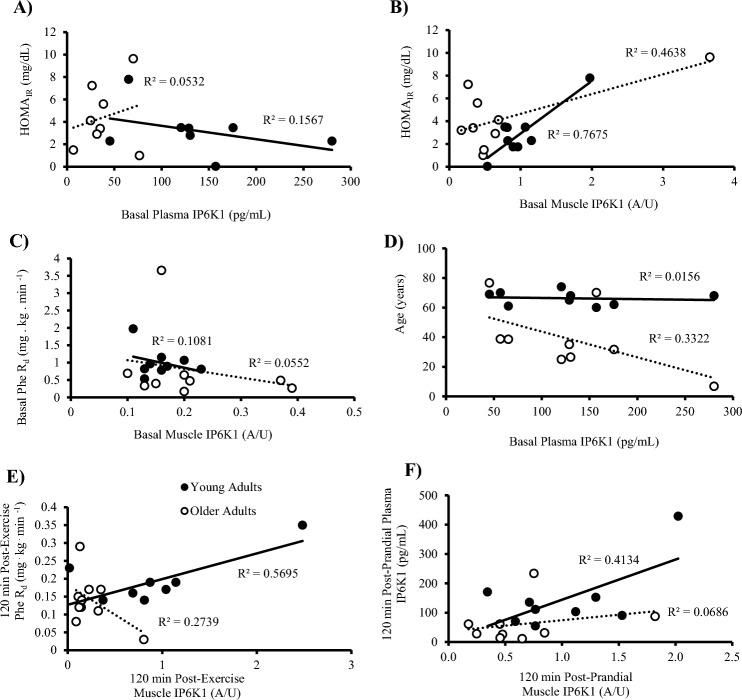
Correlation analysis for young (*n* = 9) and older (*n* = 9) adults in the post-absorptive (basal) state for young and older adulats are displayed for (**A**) HOMA_IR_ & plasma IP6K1 (Young, *P* = 0.090; Older,* P* = 0.299), (**B**) HOMA_IR_ & muscle IP6K1 (Young,* P* = 0.002; Older, *P* = 0.042), (**C**) phenylalanine rate of disappearance (R_d_) & plasma IP6K1 (Young, *P* = 0.895; Older, *P* = 0.297), (**D**) age & plasma IP6K1 (Young, *P* = 0.653; Older, *P* = 0.444). (**E**) 120 min Post-exercise phenylalanine rate of disappearance (R_d_) & muscle IP6K1 (Young, *P* = 0.019; Older, *P* = 0.148). (**F**) 120 min Post-prandial plasma IP6K1 & muscle IP6K1 (Young, *P* = 0.062; Older, = 0.496).

In the post-absorptive state, HOMA_IR_ was positively correlated with muscle IP6K1 (*r* = 0.641, *P* = 0.012) but not with plasma IP6K1 (*r* = -0.225, *P* = 0.112) (Fig. 6). In the same state, plasma IP6K1 concentration was negatively correlated with age (*r* = -0.640, *P* = 0.008). Muscle IP6K1 was positively correlated with total phenylalanine R_a_ (*r* = 0.522, *P* = 0.026), MCR (*r* = 0.725, *P* = 0.001) and plasma IP6K1 (*r* = 0.519, *P* = 0.039) at 120 min post-prandial. At 240 min post-prandial, plasma IP6K1 was negatively associated with age (*r* = -0.668, *P* = 0.007) while a positive correlation was observed between muscle IP6K1 and plasma glucose concentration (*r* = 0.715, *P* = 0.01). Post-exercise (120 min) total phenylalanine R_d_ was positively correlated with muscle IP6K1 (*r* = 0.482, *P* = 0.043) but the correlation with plasma insulin concentrations was negative (*r* = -0.647, *P* = 0.021). At the same time point, plasma IP6K1 was positively correlated with p/tAkt^473^ (*r* = 0.487, *P* = 0.047) and negatively correlated with age (*r* = -0.653,* P* = 0.004), whilst muscle IP6K1 also demonstrated an inverse relationship with age (*r* = -0.487, *P* = 0.04). Finally, at 240 min post-exercise total phenylalanine R_a_ (*r* = -0.618, *P* = 0.006), R_d_ (*r* = -0.557, *P* = 0.016) and plasma IP6K1 (*r* = -0.633, *P* = 0.009) offered an inverse correlation with age.

## Discussion

This is the first in vivo study to characterise and compare IP6K1 in the post-absorptive, post-prandial and post-RE states in young and older insulin-resistant humans. Contrary to our hypothesis and our previous findings, the current older insulin-resistant cohort (HOMA_IR_: 3.2) displayed a blunted IP6K1 response to exercise compared to the young. The current results showed that young adults had greater post-absorptive plasma IP6K1 compared to insulin-resistant older adults. In addition, there was no effect of protein ingestion or protein + RE in either group . In skeletal muscle, young adults displayed decreased post-prandial IP6K1 at 240 min compared to 120 min and basal within group. It was hypothesised that RE would decrease IP6K1 given previous findings^1^. At 120 min post-exercise muscle IP6K1 was elevated in young versus older adults which suggests IP6K1 may be involved in the anabolic response to RE in the young insulin-sensitive but seems to be blunted in skeletal muscle in the older insulin-resistant group. Its worth highlighting that the current study is limited in that it cannot dissect the differences between insulin resistance and the ageing process. Financial limitations prevented an additional old, insulin sensitive group. In addition, if there was a greater amount of muscle tissue available, we would have liked to explore additional protein targets specifically implicated in senescence signalling (i.e. myostatin-SMAD2/3 signalling)^17^, as well as more targets implicated in insulin signalling (i.e. p70^s6k^ and IRS-1) to explore IP6K1’s role, in this pathway, further. Here we showed that older adults presented with a reduction in total phenylalanine R_d_ versus young adults, paired with elevated circulating insulin in the post-exercise/post-prandial states^18^. Taken together, these data suggests that the reduction in muscle and plasma IP6K1 may have a role to play in the insulin-mediated responses to both protein ingestion and RE, supporting its possible role in insulin-resistance.

We have previously shown that muscle IP6K1 is associated with insulin resistance in pre-diabetic middle-aged adults^2^ and muscle IP6K1 is increased in obese, insulin-resistant adults after the ingestion of a protein meal^7^. IP6K1 adds a pyrophosphate motif to generate IP_7_ which then binds to the PH domain of Akt thus preventing translocation to the cell membrane and phosphorylation by PDK1^19^. This negative effect on Akt’s has been previously shown to impair insulin sensitivity and amino acids metabolism in obese, pre-diabetic and T2Ds^2,3,7^. Here, we show that muscle IP6K1 does not increase after the ingestion of 25 g whey protein isolate in young or older adults. Moreover, basal muscle IP6K1 offered a positively relationship with HOMA_IR_ (data not shown) (*r* = 0.641, *P* = 0.012) which is consistent with a previous observation^2^. Plasma insulin was not correlated with either muscle or plasma IP6K1 for all conditions, suggesting that concentrations of IP6K1 may not be dependent on circulating insulin irrespective of age. Our previous research showed that IP6K1 muscle content was elevated at 300 min in obese insulin-resistant middle-aged adults (HOMA_IR_: 5.8) during a post-prandial period (36 g lean protein), a finding not replicated in lean otherwise matched controls here. Moreover, there was no difference in skeletal muscle IP6K1 between young and older adults throughout the basal and post-prandial period which suggests obese insulin-resistant adults respond differently to older adults who present with insulin resistance (HOMA_IR_: 3.2)^7^. Given our data shows elevated muscle IP6K1 does not inhibit Akt signalling, it appears IP6K1 may reach an intracellular threshold, higher than demonstrated in this study, whereby Akt is then dysregulated via PIP3 at the PH domain of Akt^1^. Thus, it can be concluded that the young adult response to a protein feed may involve a reduction in muscle IP6K1 over a 2–3 h period which may contribute phenylalanine disposal in a healthy young insulin-sensitive state.

We hypothesised that RE would also reduce IP6K1 while elevating pAkt in young and older adults, leading to amplification in intracellular anabolic signalling. This data showed that older adults had significantly less muscle IP6K1 120 min post-exercise compared to young controls, however there was no difference at 120 min post-exercise versus basal in either group, suggesting that muscle IP6K1 was not responsive to RE or that the RE exercise stimulus employed was not potent enough to provoke a change in this pyrophosphate kinase Its worth noting that the present study design was constructed to understand the differences in IP6K1 between young and older adults in the context of MPS. Thus, we may have missed potential differences in muscle IP6K1 that a time course design may have drawn out. However, post-exercise phenylalanine R_d_ was positively linked with muscle IP6K1 yet showed a negative relationship with plasma insulin. This suggests that IP6K1 may be involved in the normal response to exercise as seen in the young and moderately active control group, similar to the above post-prandial IP6K1 evidence. Previous research investigating pre-diabetics showed elevated IP6K1 was associated with hyperinsulinemia and insulin resistance^2^. In the current study, elevated muscle IP6K1 was not associated with a modest increase in HOMA_IR_ which suggests this mechanism may be insulin-resistant dependent, and that IP6K1 role may differ in aging when compared to pre-diabetic skeletal muscle. Moreover, a single bout of aerobic exercise had no effect on muscle IP6K1, whereas a time-matched repeated sprints exercise resulted in an intramuscular decrease in IP6K1^2^. However, it should be noted that this effect was noted in pre-diabetic individuals who presented similar HOMA_IR_ (3.3)^2^ compared to the current study. Taken together, it appears that IP6K1 response to exercise is intensity or mode dependant. Yet, we acknowledge that there is currently little evidence to support this statement. Moreover, ageing muscle (66 ± 5 years) may still be more insulin responsive compared to late middle-aged diabetic/pre-diabetic muscle (47 ± 3 years).

This research is the first to compare plasma IP6K1 in young and older adults. In contrast to our hypothesis, older adults had lower plasma IP6K1 concentration compared to young controls (Fig. 2A). The only study to date that has investigated plasma IP6K1 suggested that there is a positively relationship with HOMA_IR_ which was not seen in the current study. AUC for insulin and glucose were not different between groups; however, HOMA_IR_ was significantly increased in the older adult group, yet lower than previously published in pre-diabetics^2^. It has been shown that the IRS-1 and IGFR signalling operate interchangeably to facilitate cell growth and metabolism^5^. In the current study, older adults had greater basal IGFR compared to younger adults. IRS-1 is a known stimulator of muscle IP6K1 via PIP_2_^3,19^. Given the reduced IGFR activity in the young sample, it is reasonable to postulate that IRS-1 may be providing a compensatory mechanism to increase Akt and 4E-BP1 phosphorylation^5^. Furthermore, IRS-1 is known to increase in the post-exercise state in young adults^20^. Moreover, young adults demonstrate increased p-4E-BP1^37/46^ in response to anabolic stimuli, which is often paired by an increase in p-p70^s6k^. Both p70^s6k^ and 4E-BP1 are vital for the initiation and accuracy of protein translation across the ribosome, and the reduction seen in older adults will be an attenuating factor in the skeletal muscle ageing process^21–23^. Increased p70^s6k^ and IP6K1 have been seen previously in two separate studies^20,24^. In previous efforts, people with obesity had increased post-prandial p70^s6k^^24^, IGFR and IP6K1 protein content^7^, which is different to the older adult characteristics in the current study who showed reduced IGFR versus young adults. Taken together, this data suggests that IP6K1 may play an important role in young insulin-sensitive adult’s ability to dispose and utilise amino acids as supported by the total phenylalanine R_d_ results. It is thought that the increase in plasma IP6K1 in young adults may be a direct result of increased muscle receptor activity due to increased p70^s6k^ activity seen in a previous effort using the same exercise stimulus^25^. However, it is noted that p70^s6k^ and IRS-1 are missing from the data set due to the sample being exhausted for muscle isotope enrichment analysis. Finally, the evidence presented indicates that obese and older adult’s anabolic resistance may differ depending on the degree of metabolic dysfunction^26^.

Given the evidence outlined above, we proposed that any increase in IP6K1 would likely decrease p/tAkt^308^ and p/tAkt^473^, therefore decreasing downstream indicators of MPS (i.e., p-4E-BP1^37/46^). In this study, RE and protein ingestion increased p-Akt^308^ in young adults (Fig. 4B). Similarly, young adults saw an increase of p-Akt^473^ at 240 min post-exercise versus basal (Fig. 4D). The current findings indicated that muscle p/tAkt^308^ and p/tAkt^473^ were all similar between groups, suggesting that IP6K1 may not have had any negative effect on Akt activity in the IGF-1 signalling paradigm. Thus, the increase of p-4E-BP1^37/46^, which is vital for relieving its inhibitory effect on eIF4E^23^ and initiating mRNA translation, may not be completely reliant on Akt-mTORC1^27^. This Akt-independent mechanism was recently found in a rodent Akt knockdown model^27^, highlighting a possible compensatory mechanism that increases MPS when Akt is disrupted. Therefore, this study presents further evidence that Akt signalling may not be pivotal for a normal anabolic response to RE.

Our results show that young adults increased total muscle LAT1 protein content at 120 min postprandial versus basal, with no between-group differences. This is supported by comparable research which saw no between-group differences in LAT1 in the post-prandial and post-exercise states between young and older adults^22^. Importantly, normal intracellular signalling of protein kinases should increase temporarily and then return to basal which has been shown to be vital for maintaining anabolic sensitivity and optimal amino acid uptake^18,21^. Previous work has suggested that increasing the protein dose per meal is a useful strategy to mitigate the resistance to anabolic stimuli in older adults by flooding the intracellular amino acid pool^18,28–30^. In the older adults of this study, a group-matched quantity of whey protein caused a reduction of phenylalanine R_d_ and muscle signalling when compared with young counterparts (LAT1, 4E-BP1 and IP6K1). Thus, the mechanisms that lead to reduced NPB in older adults surround an innate resistance to ingested amino acids at the whole-body level with a reduction in insulin responsiveness a likely contributor.

Some limitations are present in the current study are that our measurement of whole-body total phenylalanine R_d_ does not allow us to determine phenylalanine hydroxylation and thus whole-body protein synthesis and/or net protein balance cannot be determined^31^. Moreover, methodological issues related to determining muscle protein L-[ring-2H5]phenylalanine enrichments prevented us from determining muscle protein fractional synthetic rates (direct measurement of MPS). Although protein synthesis–related signaling pathways (i.e. mTORC1 siganling) are often measured as indicators of anabolic activity, they do not always directly dictate or correlate with MPS rates. Past work has shown that increases in phosphorylation of mTORC1 targets like p70S6K and 4E-BP1 do not always correspond to proportional increases in MPS^32,33^. This discrepancy occurs because single-point measurements of signaling events limit insight into the dynamics of protein synthesis and remodeling, whereas direct measurements of MPS allow for this dynamic assessment throughout a prolonged post-exercise/postprandial period.

Additionally, current methodologies make it difficult to directly measure whole-body net protein synthesis or protein balance. Traditional tracer techniques (e.g., stable isotope amino acid tracers) can assess mixed-muscle or whole-body protein turnover, but do not capture compartment-specific processes with high precision^34,35^. Muscle biopsies only provide localized snapshots of MPS rather than a comprehensive view of net protein balance across all tissues.

Thus, while signaling data are valuable for understanding molecular regulation, they should not be used in isolation as proxies for actual protein synthesis rates or net protein accretion. Integration of signaling, tracer-based MPS measurements, and whole-body protein balance approaches is necessary to accurately assess protein metabolism.’

lastly, muscle protein data was lacking both p70^s6k^ and IRS-1 due to sample exhaustion; the addition of these two proteins would have helped in understanding a potential insulin sensitivity-dependent MoA involving IP6K1.

To conclude, this study provides new evidence of a possible role for IP6K1 in normal amino acid metabolism in young and healthy adults which may be dependent on insulin sensitivity. Taken together, IP6K1 is involved in the insulin response to protein and RE however, it remains unclear how ageing, insulin resistance, insulin and IGF-1 signalling affect IP6K1 at the whole-body and molecular levels.

## Supplementary Information

Supplementary Information.

## Data Availability

All datasets generated during and/or analysed during the current study are not publicly available but are available from the corresponding author on reasonable request.

## Abbreviations

IP6K1: Inositol hexakisphosphate kinase 1
mTOR: Mechanistic target of rapamycin
mTORC1: MTOR complex 1
mTORC2: MTOR complex 2
Akt: Protein kinase B
PI3K: Phosphoinositide 3-kinases
IGF-1: Insulin-like growth factor 1
IGFR: IGF receptor
IRS-1: Insulin receptor substrate 1
Rheb, p70^s6k^: Ribosomal protein S6 kinase beta-1
4E-BP1: Eukaryotic translation initiation factor 4E-binding protein 1
MPS: Muscle protein synthesis
MPB: Muscle protein breakdown
PA: Phosphatidic acid
PtdIns3P: Phosphatidylinositol 3-phosphate
LAT1: L-type amino acid transporter 1
NPB: Net protein balance
PDK1: Phosphoinositide-dependent kinase 1
IGFBP: Insulin-like growth factor-binding protein
PH: Pleckstrin homology
BMI: Body mass index
HOMA_IR_: Homeostasis model assessment of insulin resistance
RE: Resistance exercise
1RM: One-repetition maximum
