# Supplementary figures and images for: Inositol hexakisphosphate kinase 1 is implicated in the insulin response to protein ingestion in older adults
Source: Sci Rep. 2026 Feb 18;16:9490. doi: 10.1038/s41598-026-35711-2 (PMC13005028; doi:10.1038/s41598-026-35711-2)

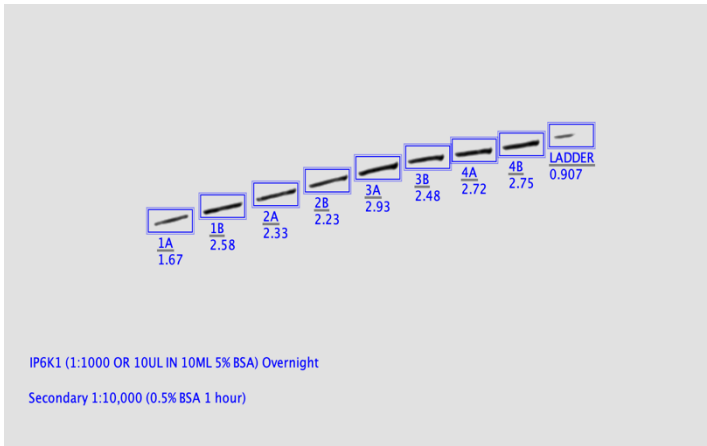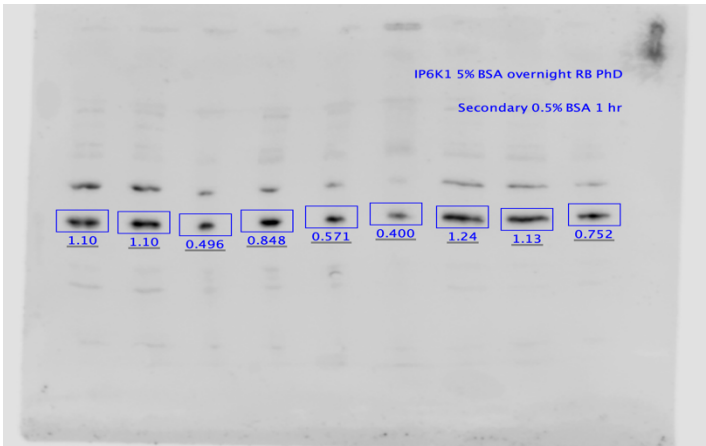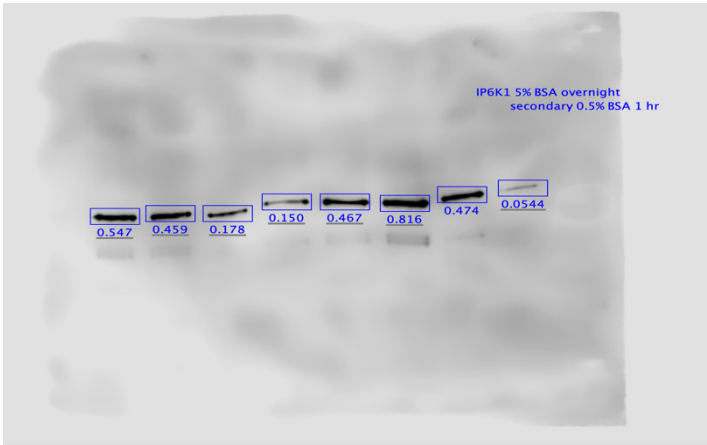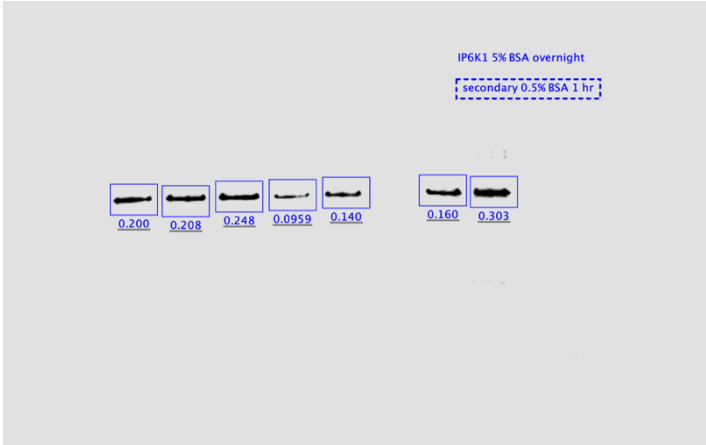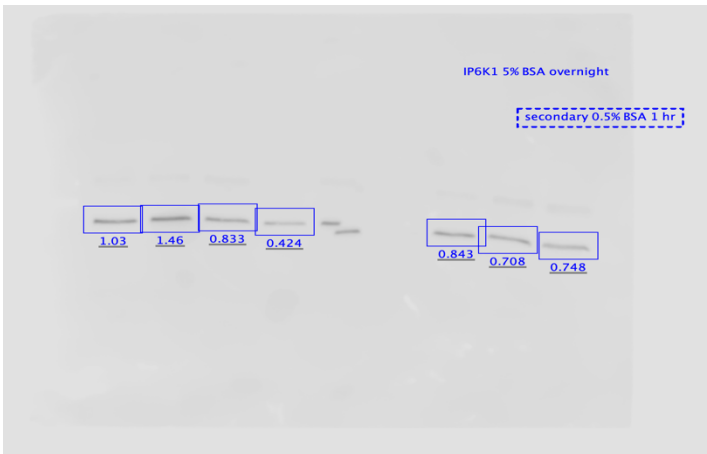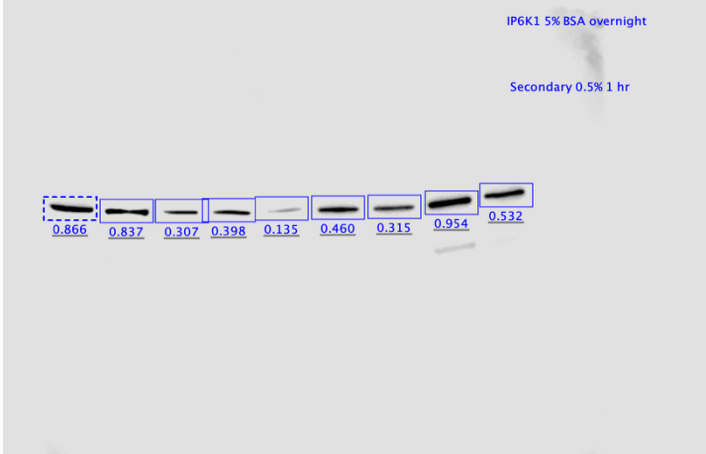

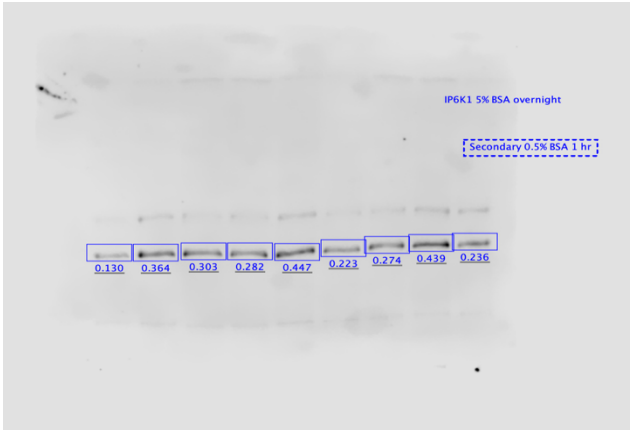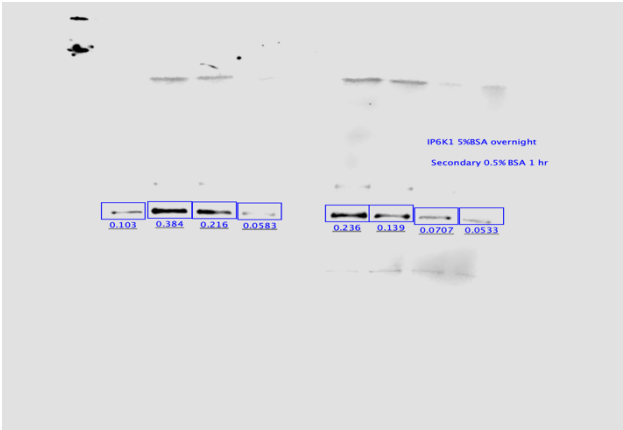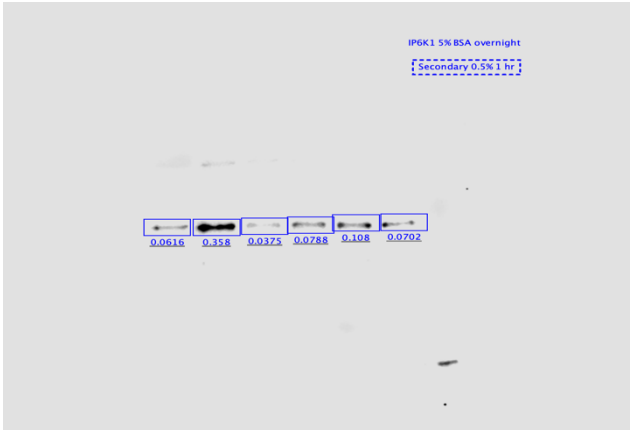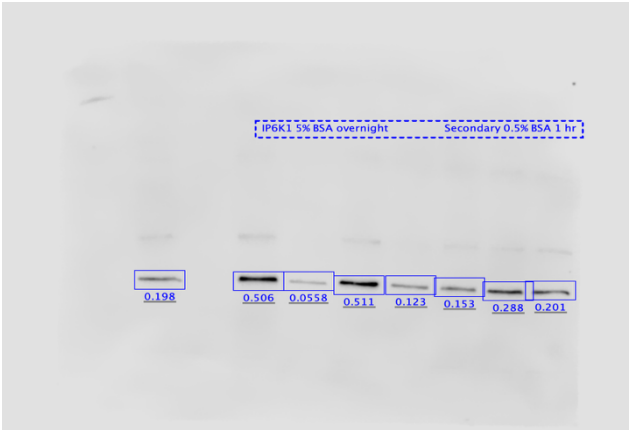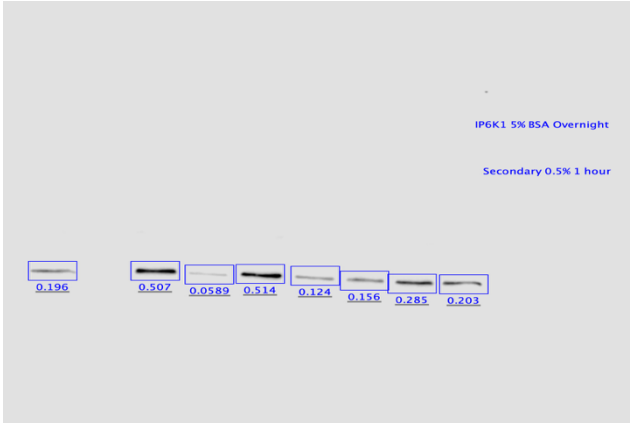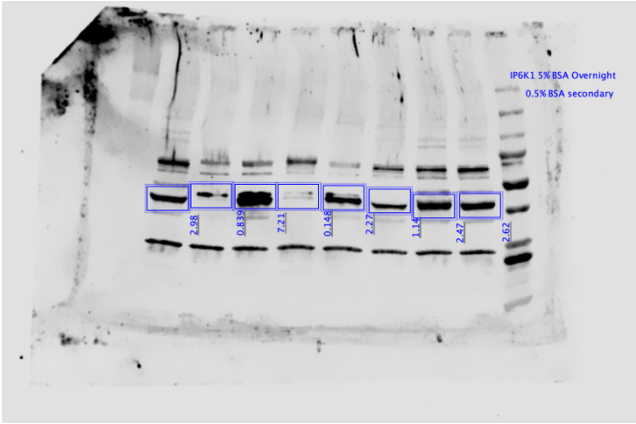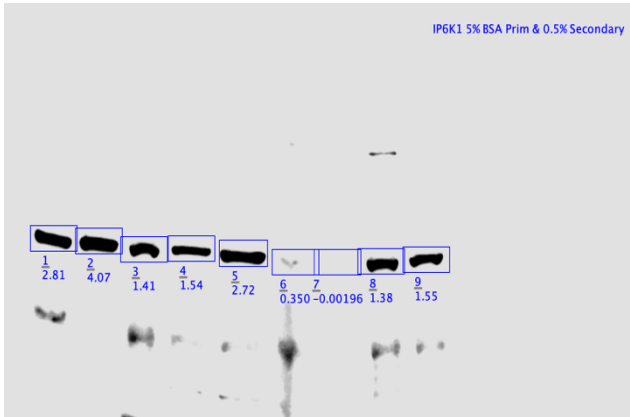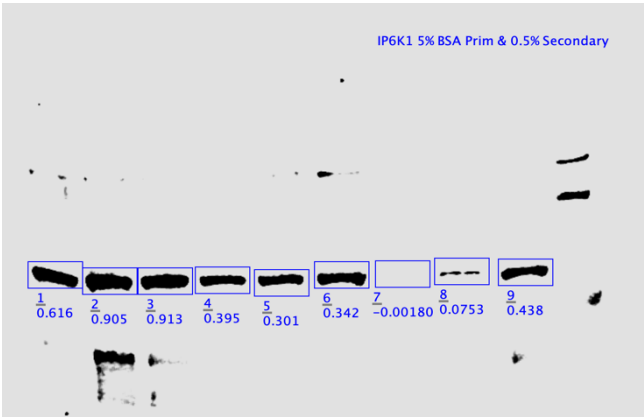

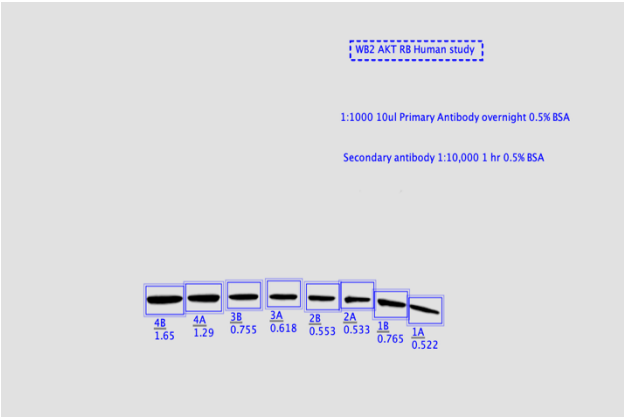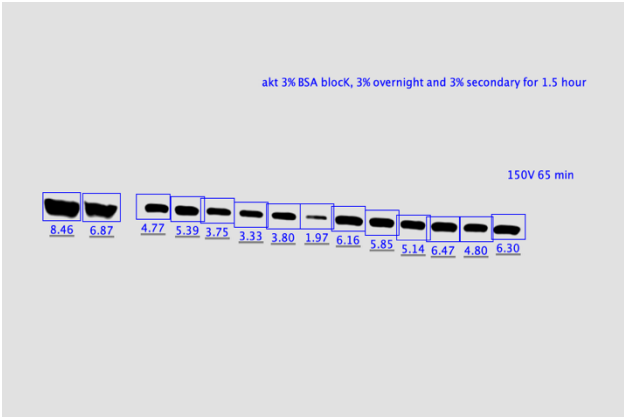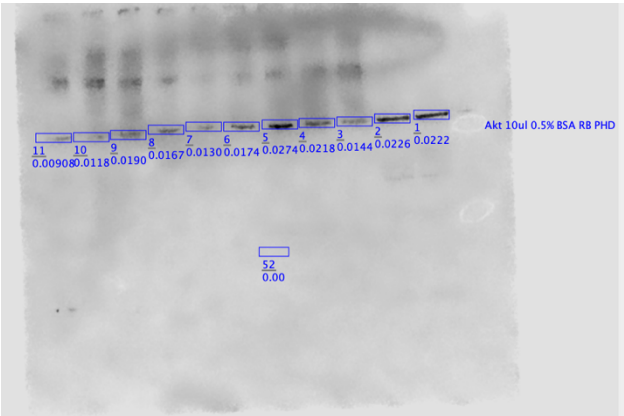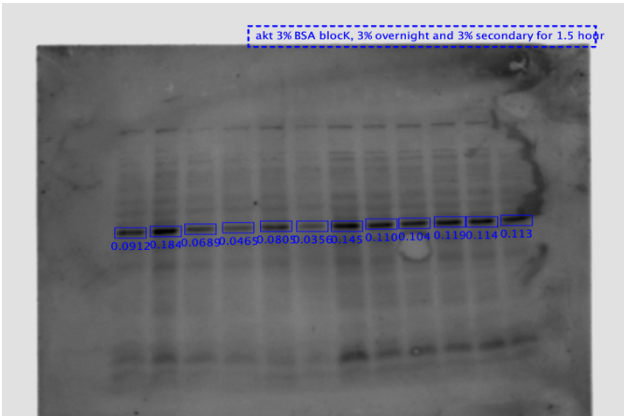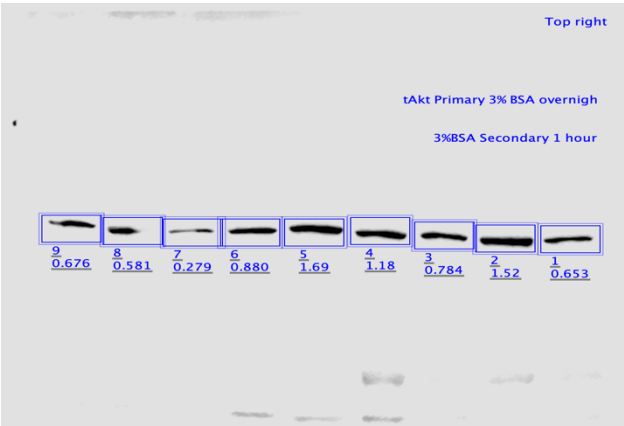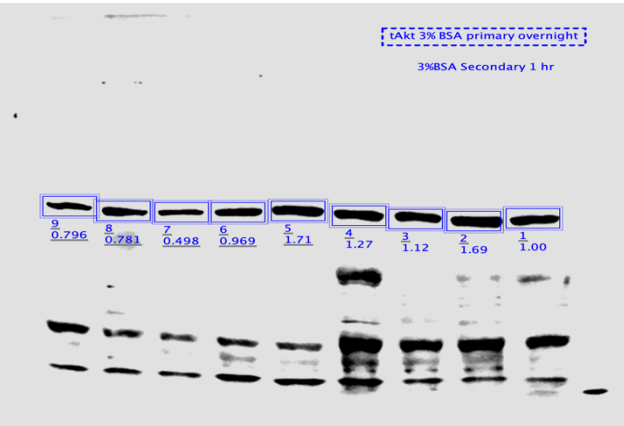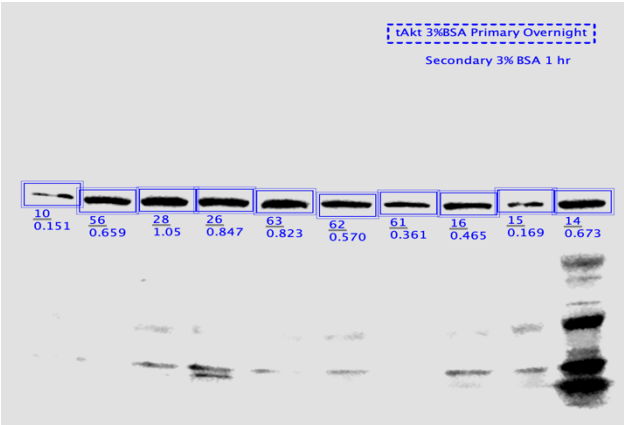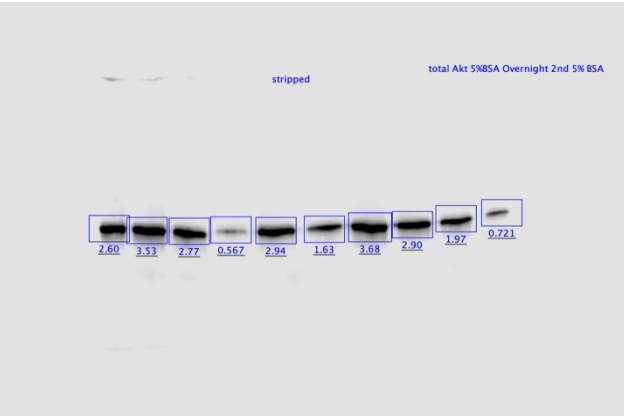

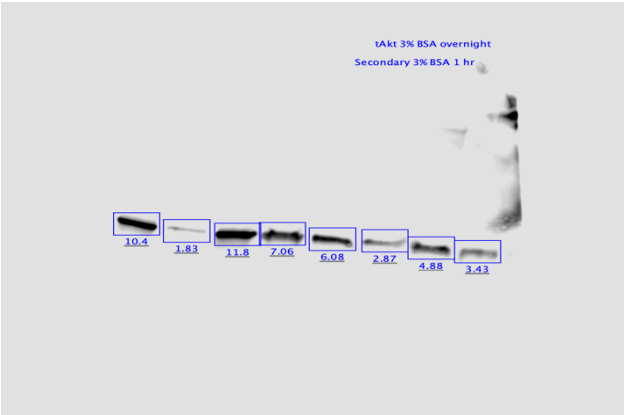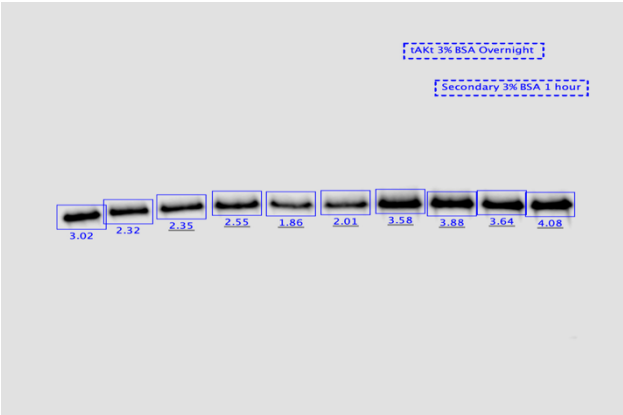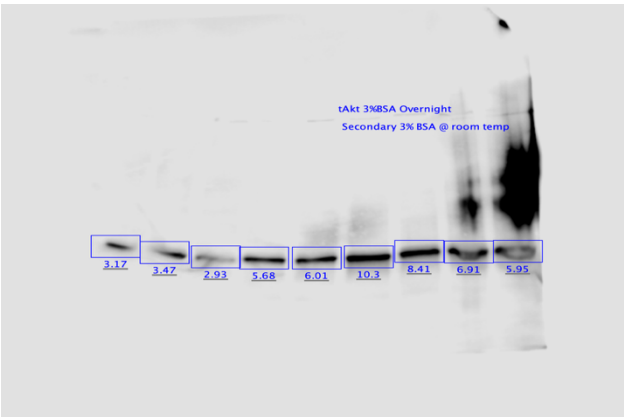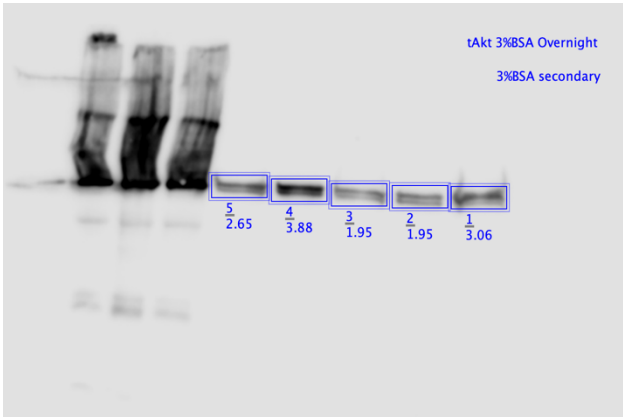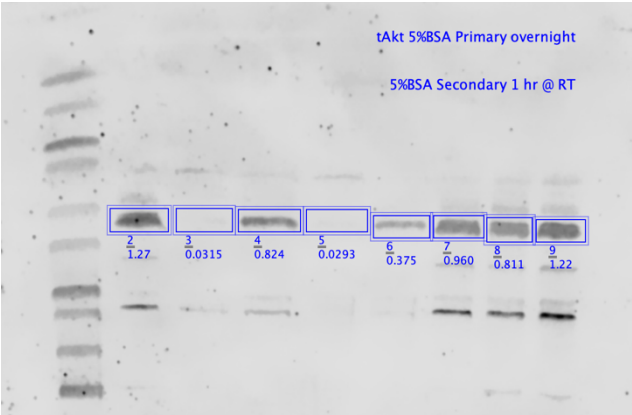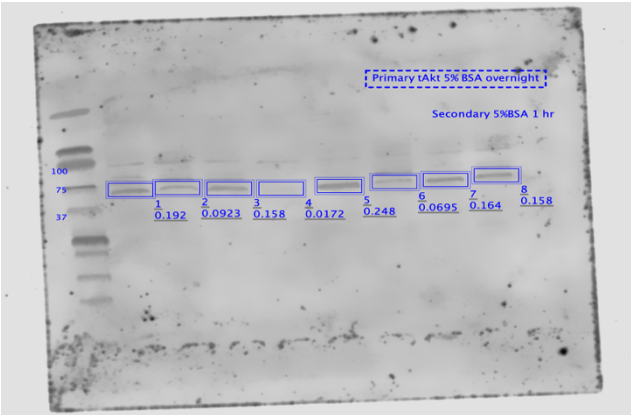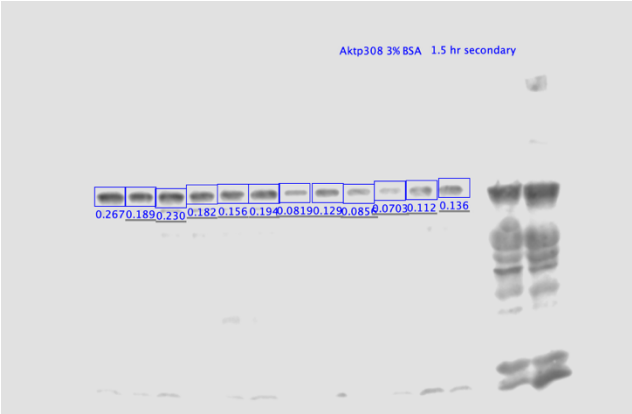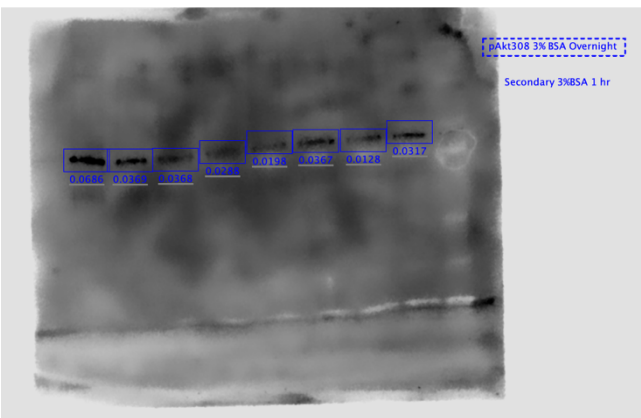

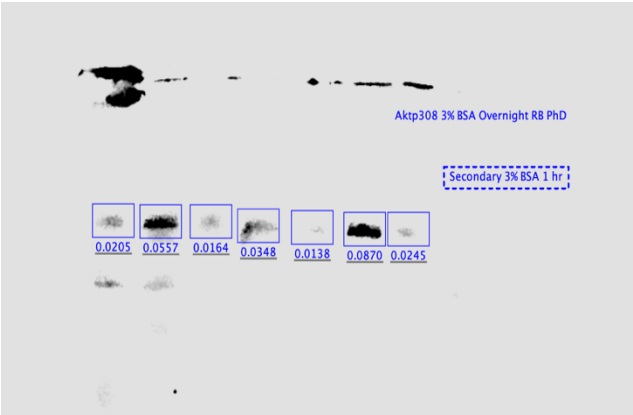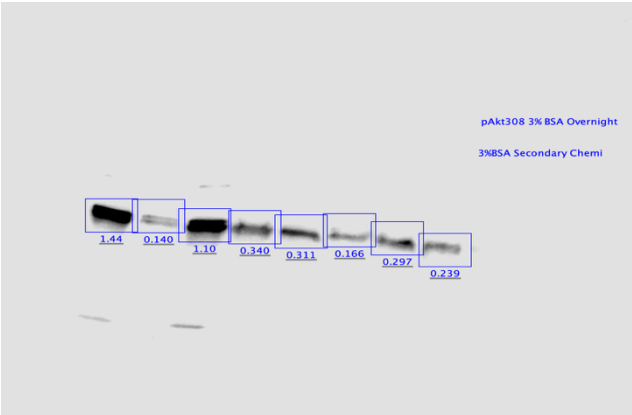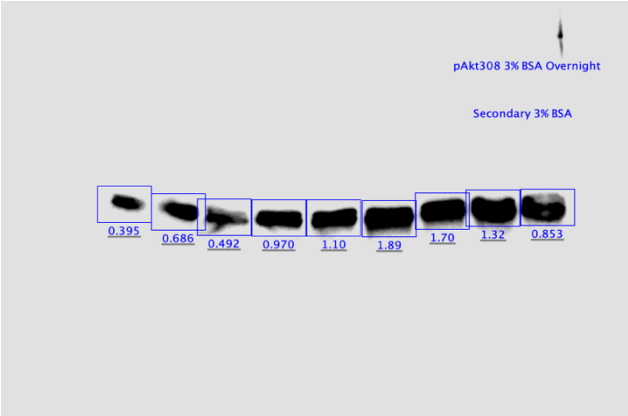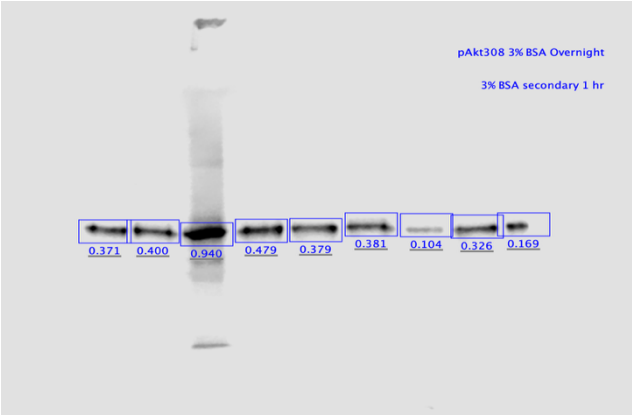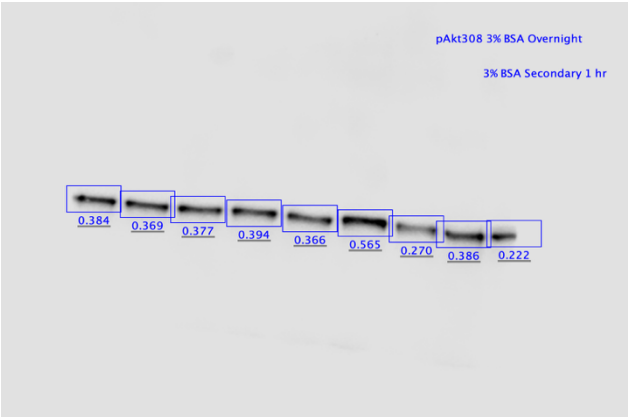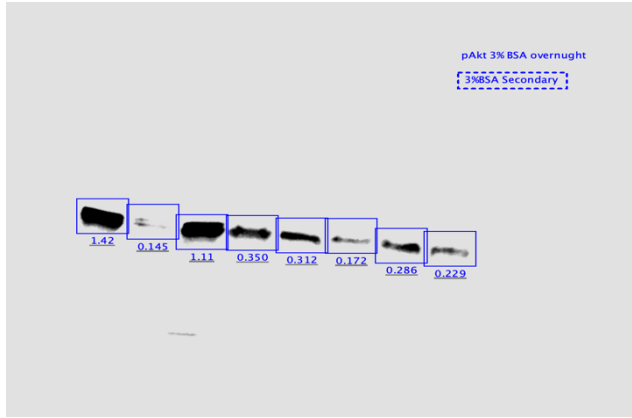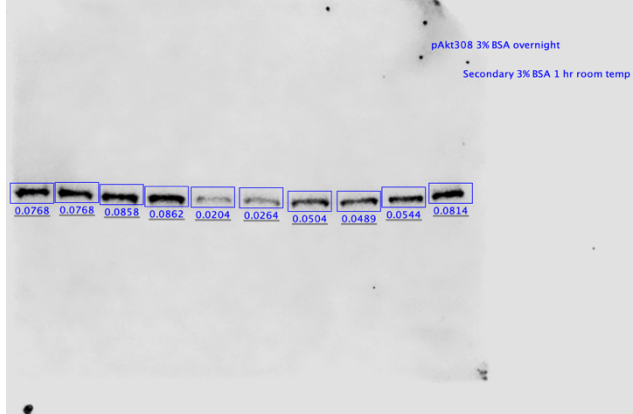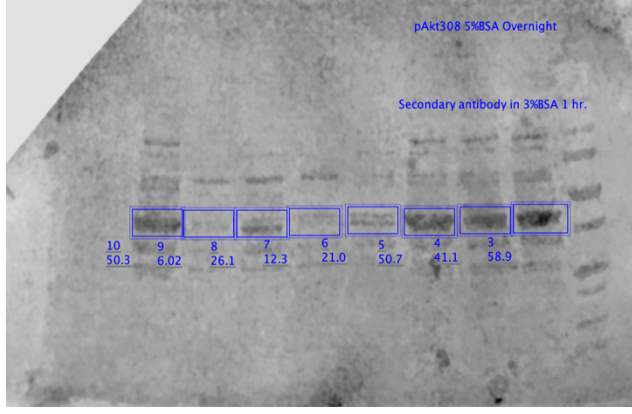

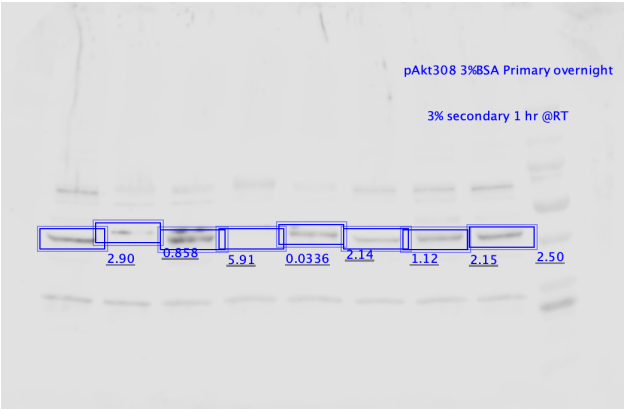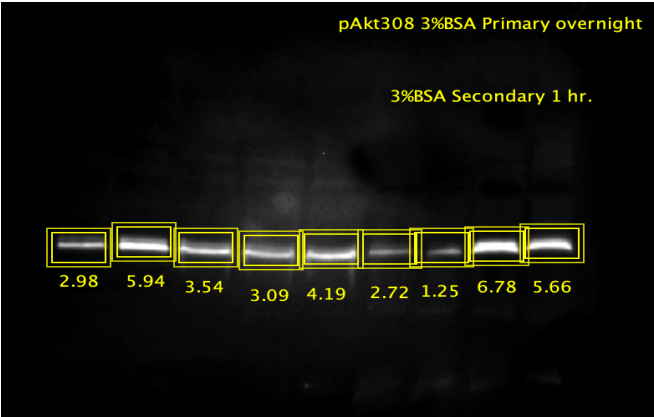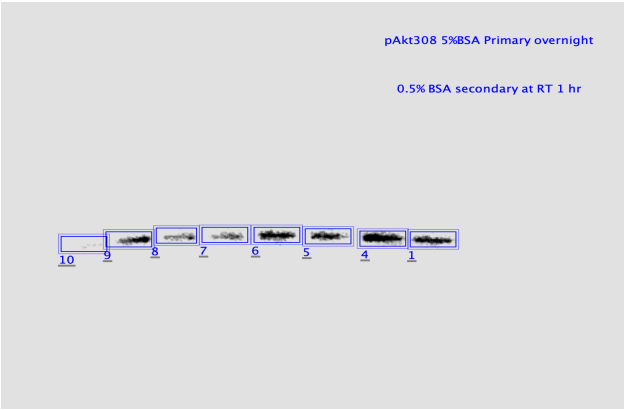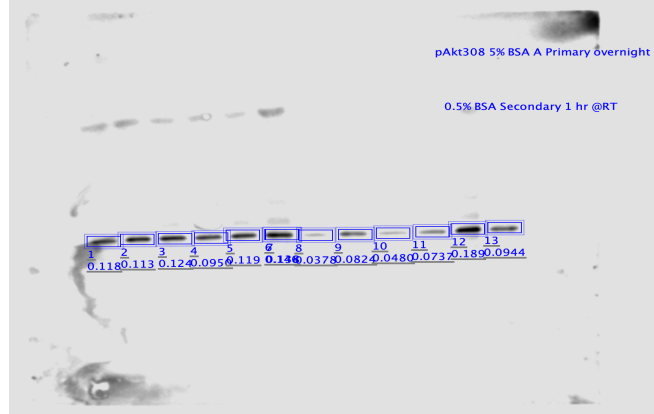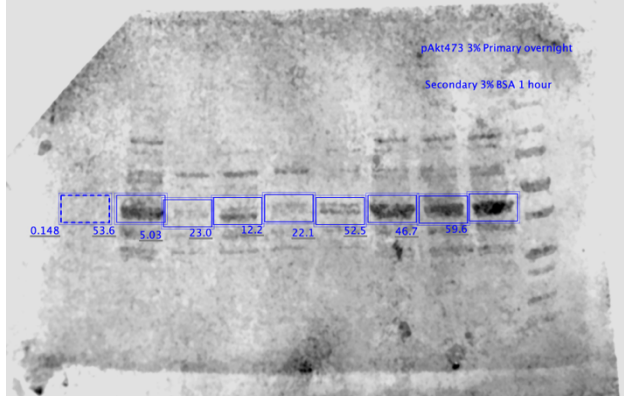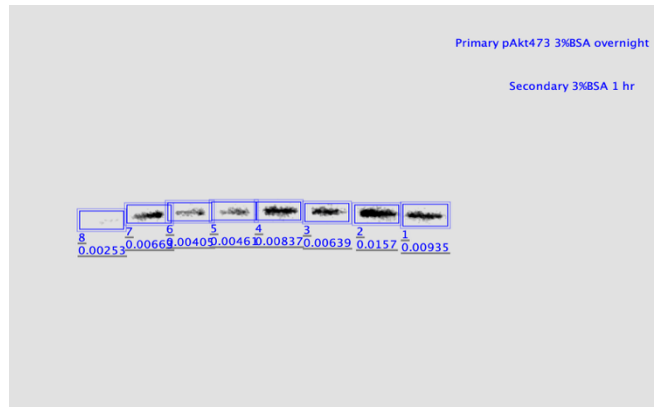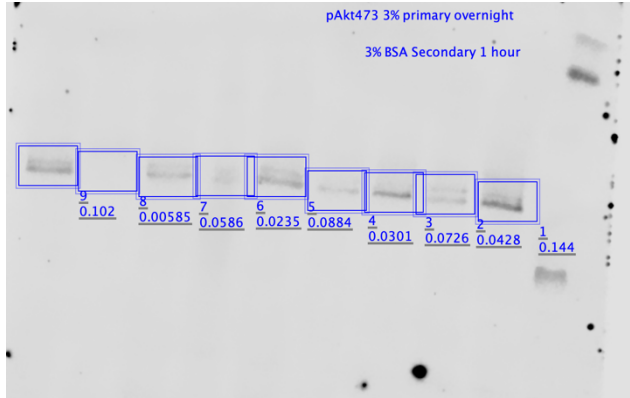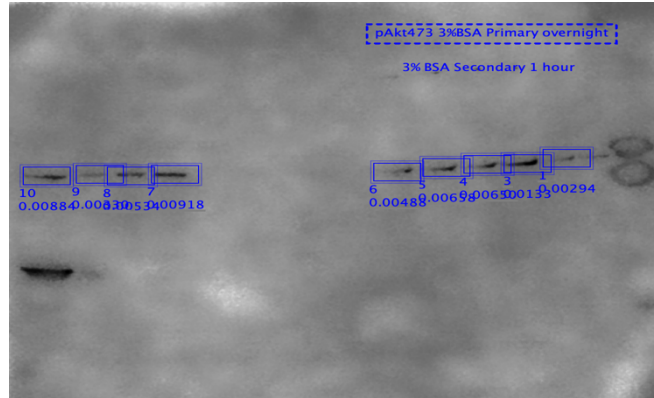

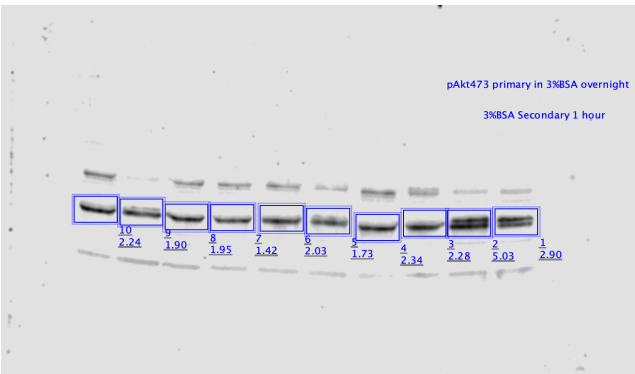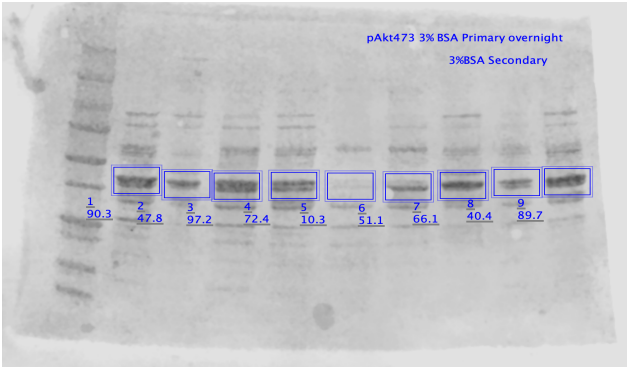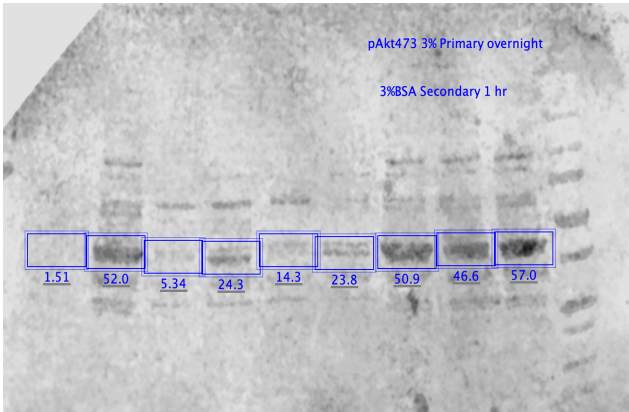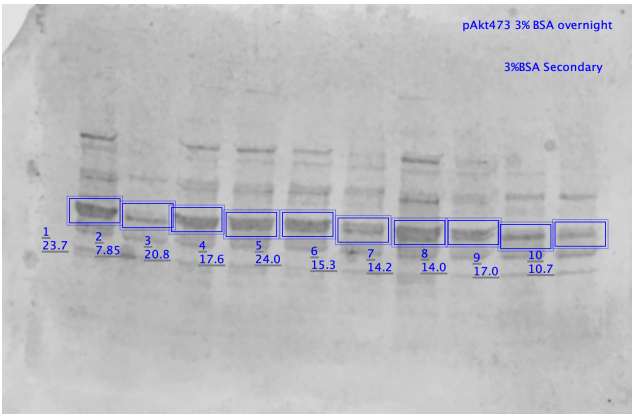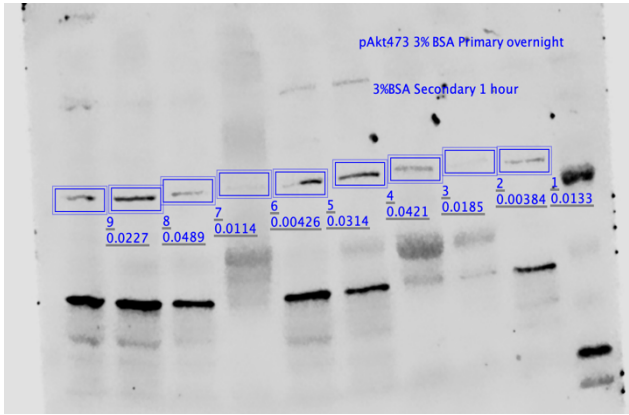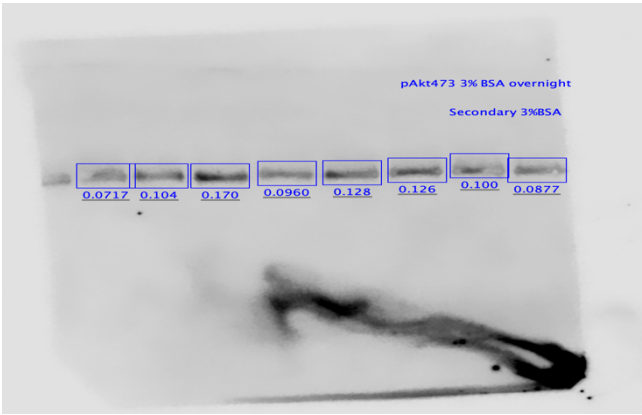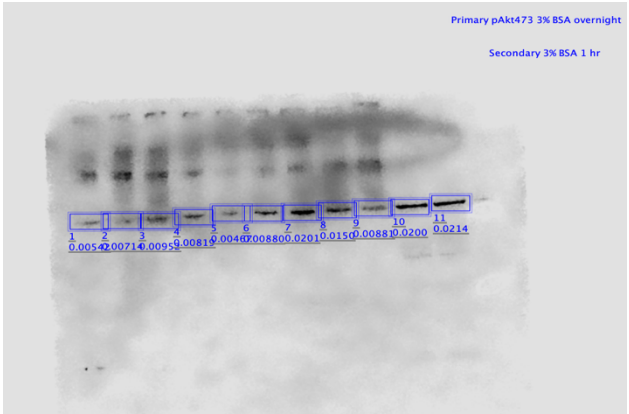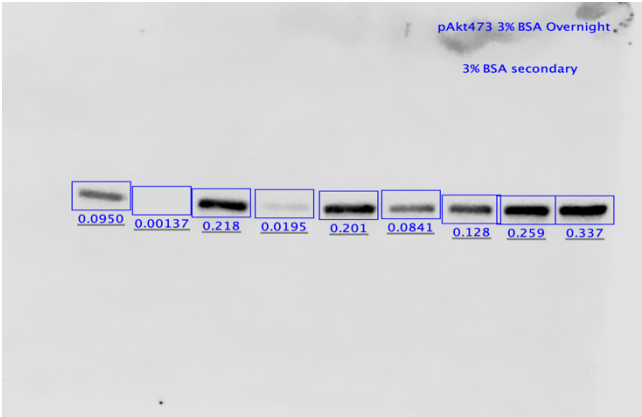

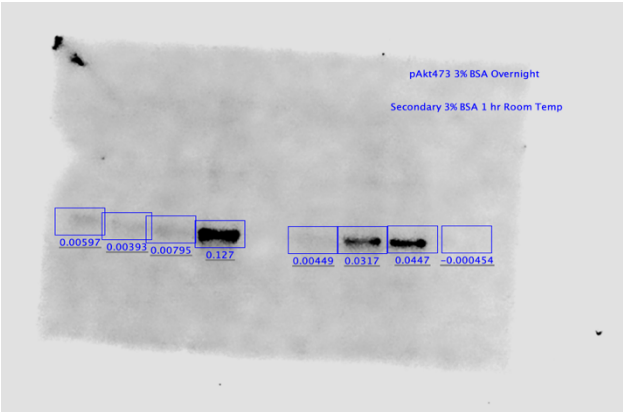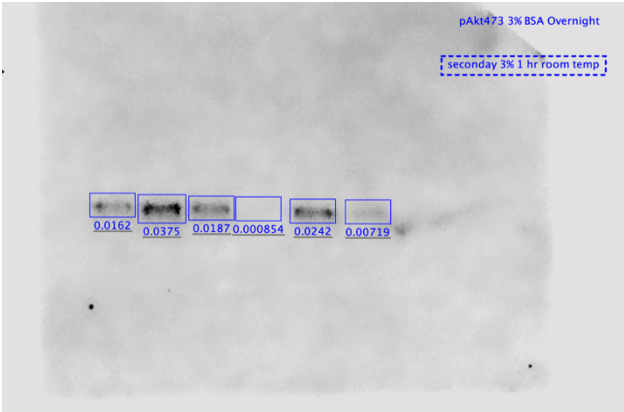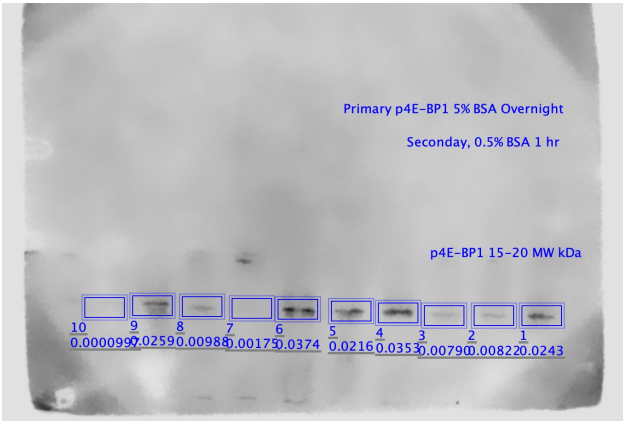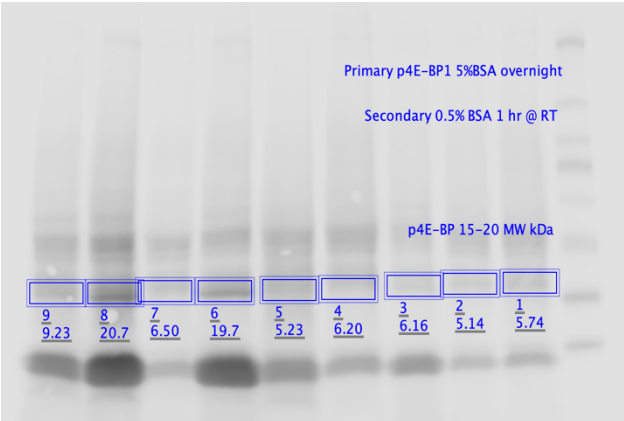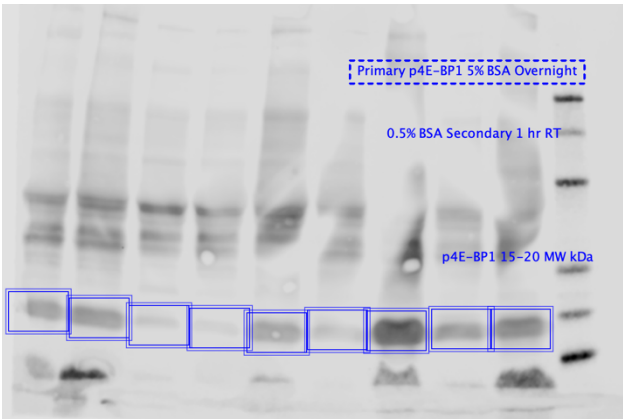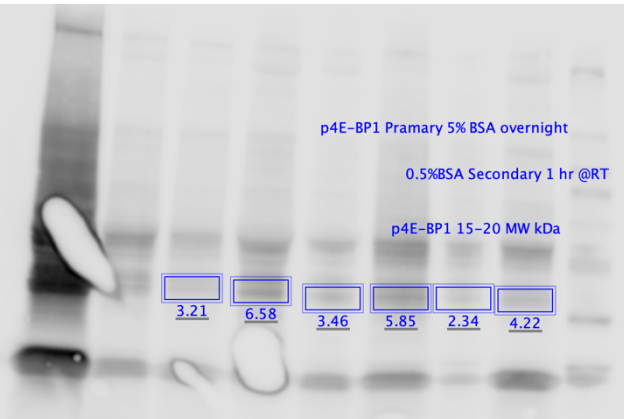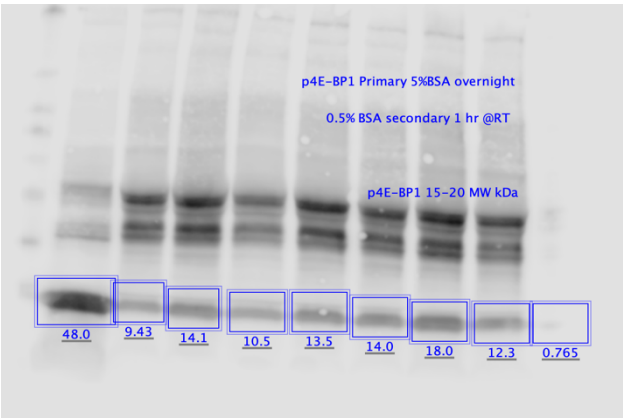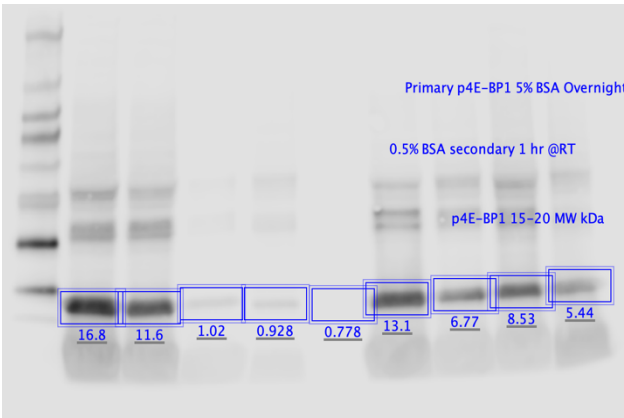

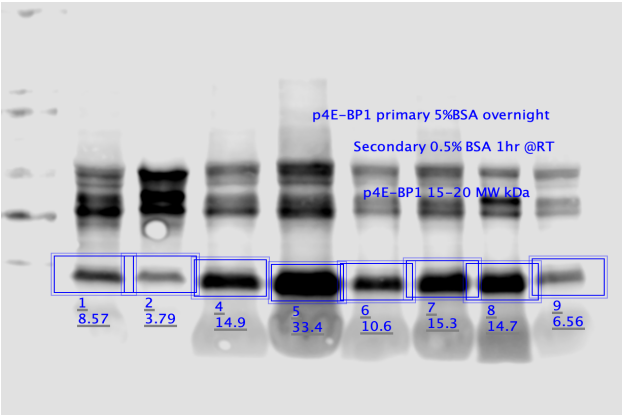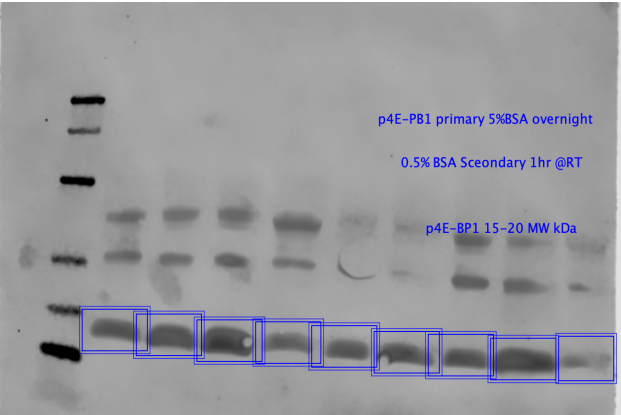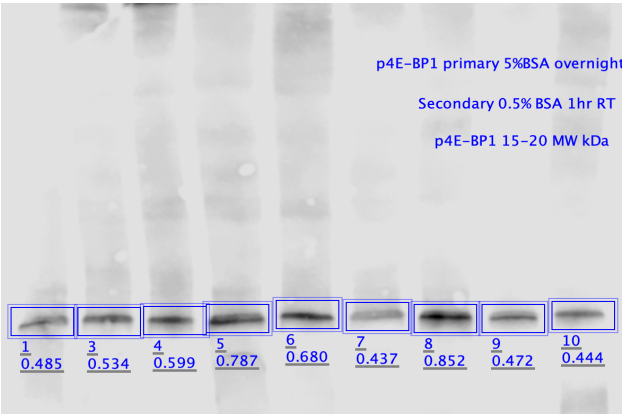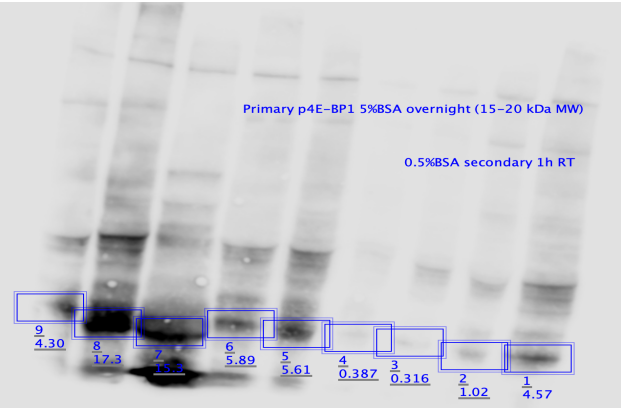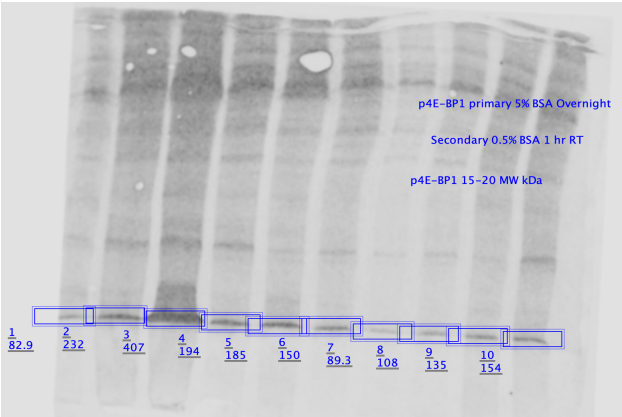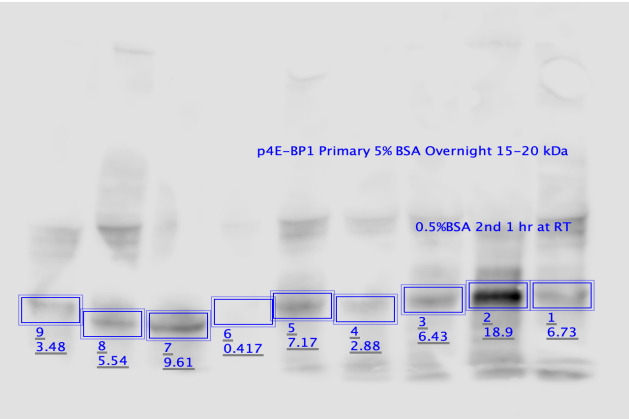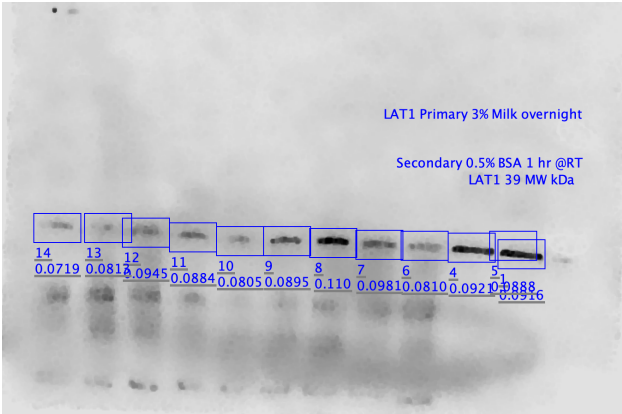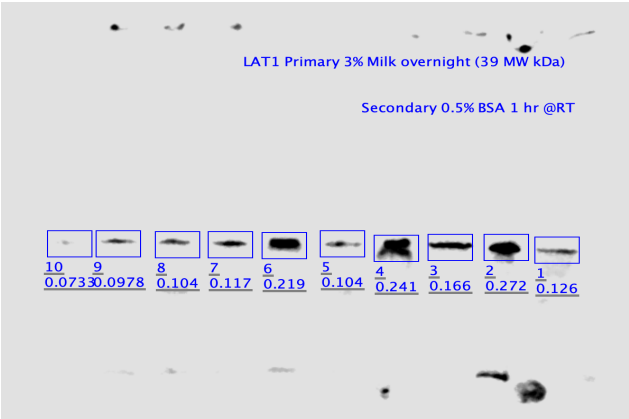

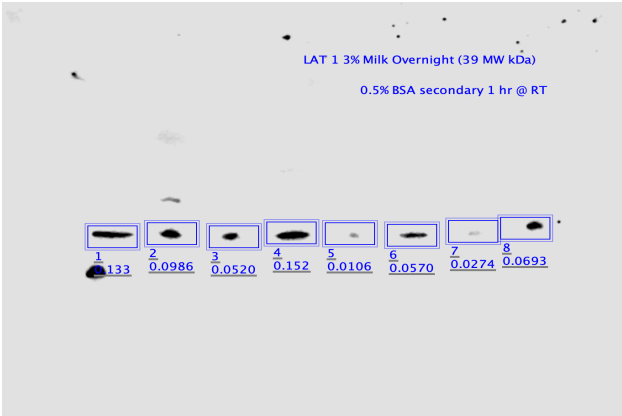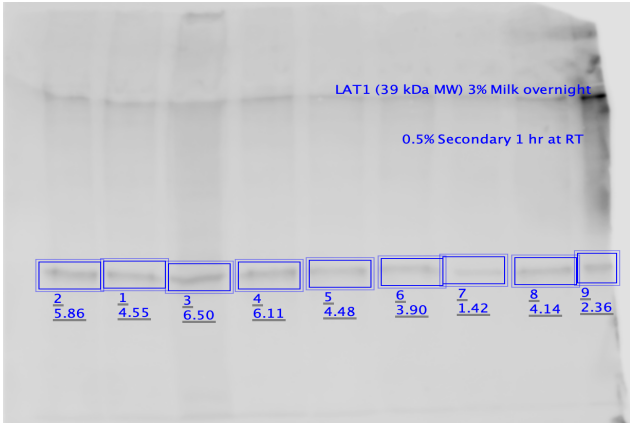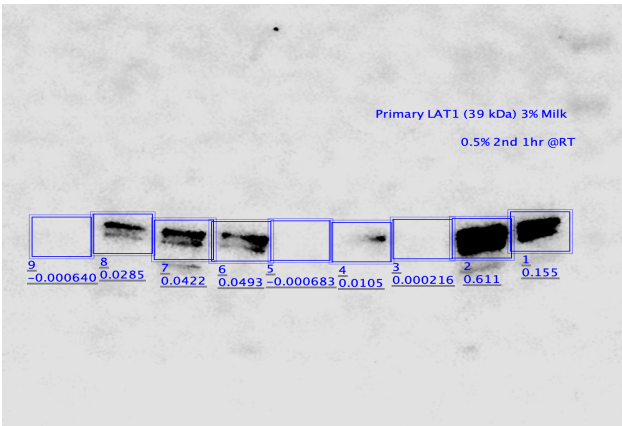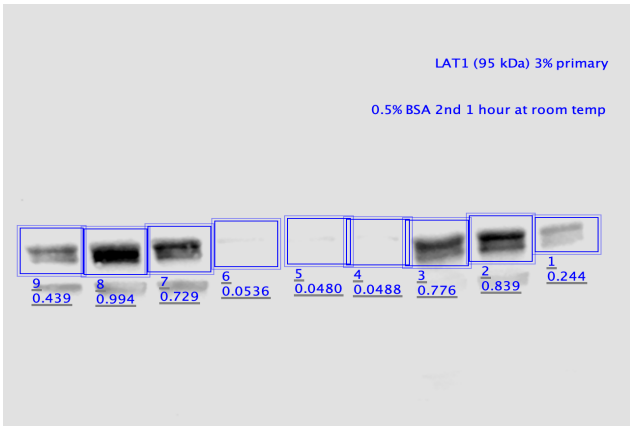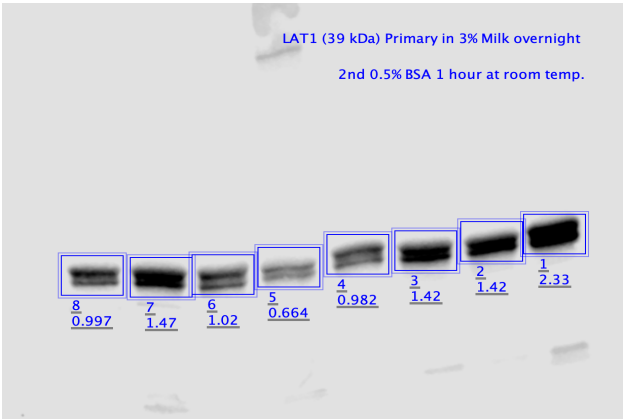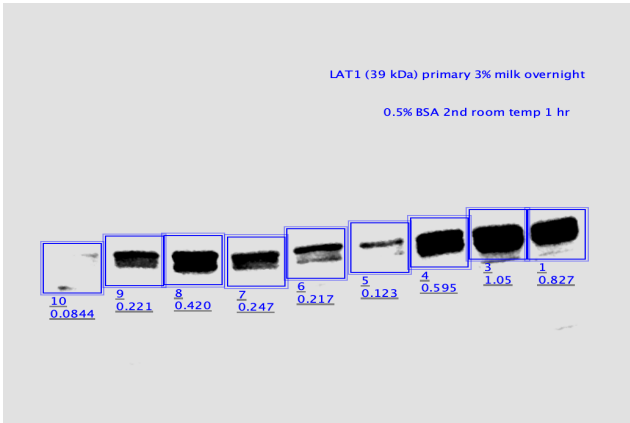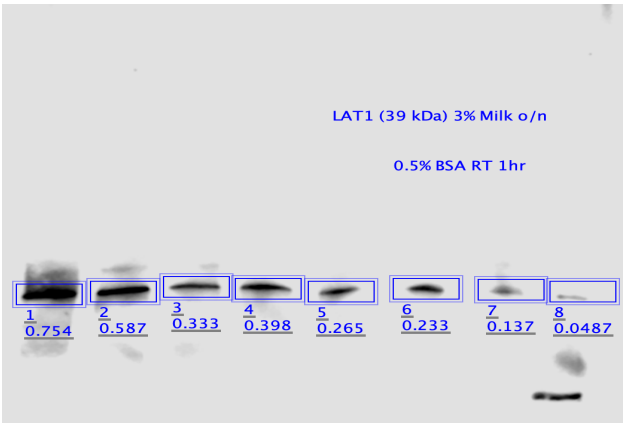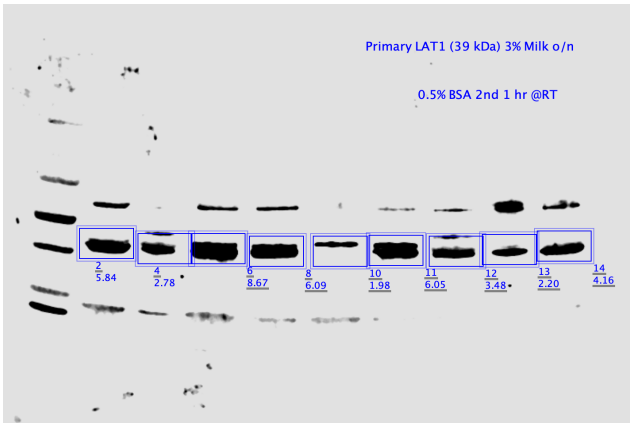

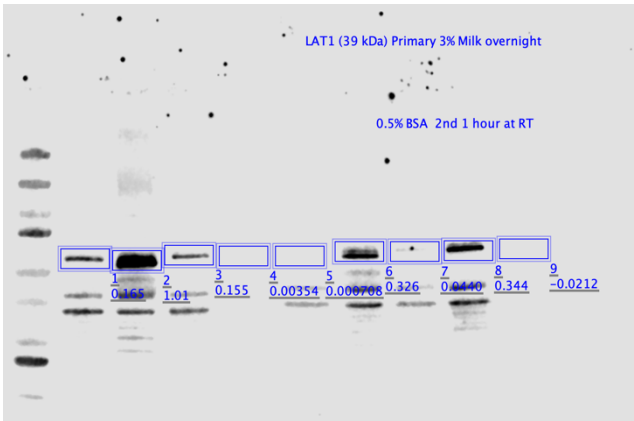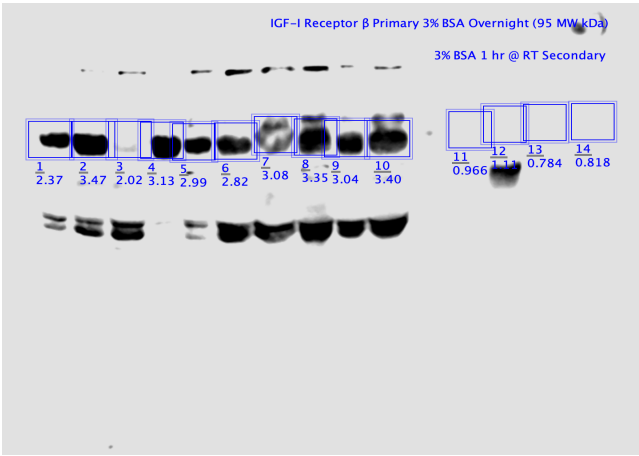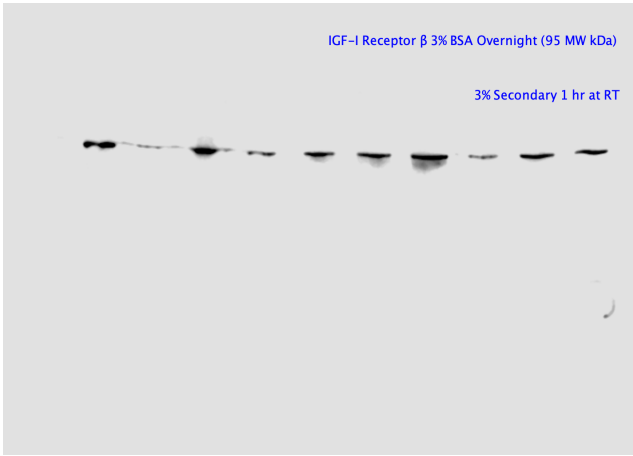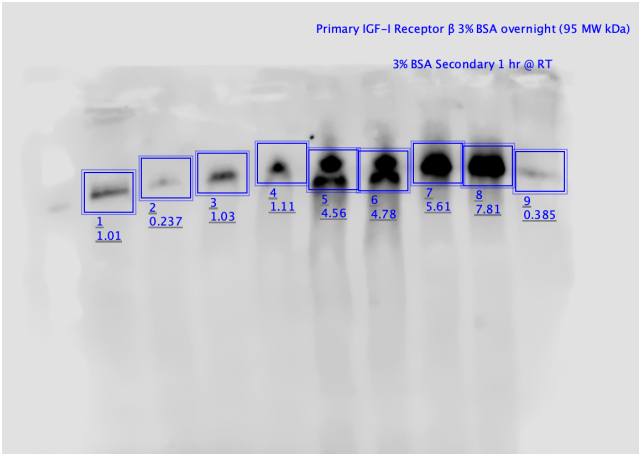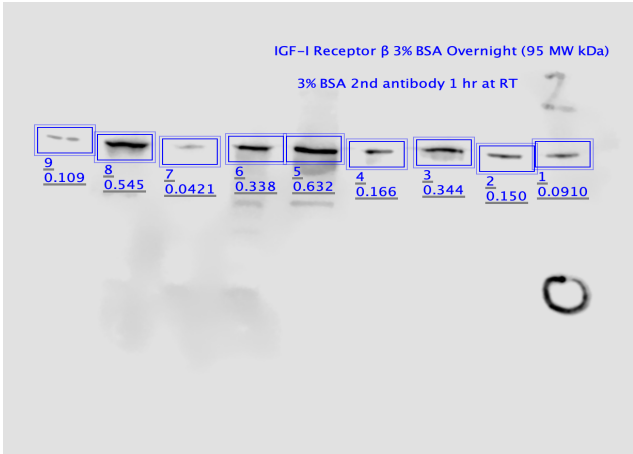

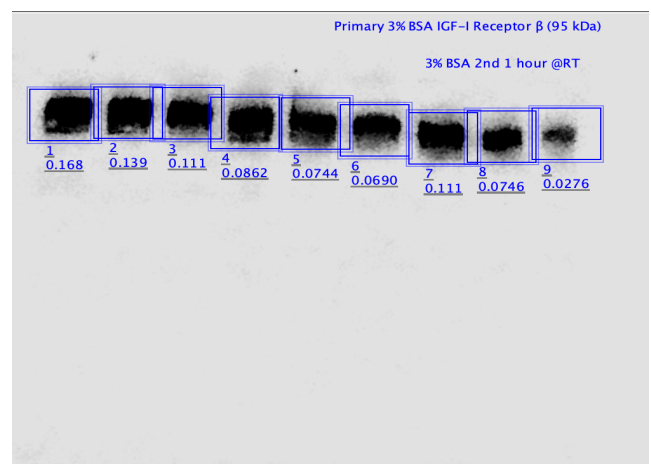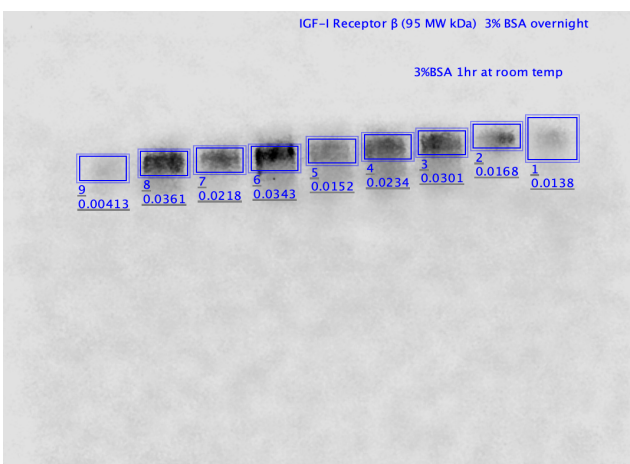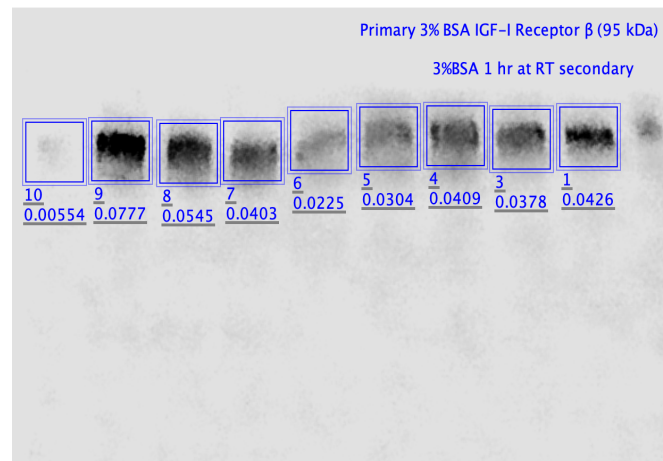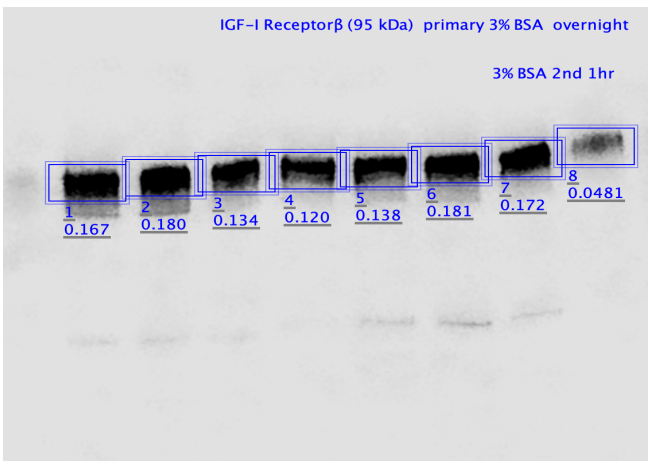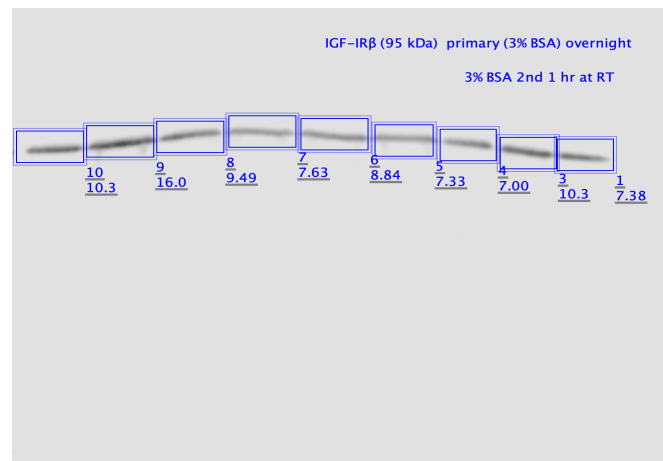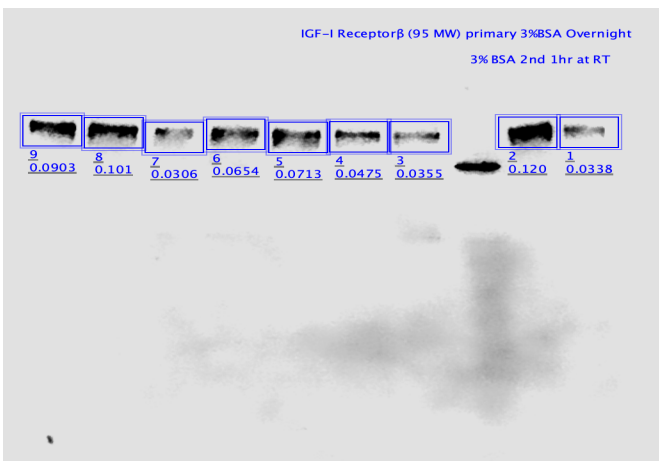

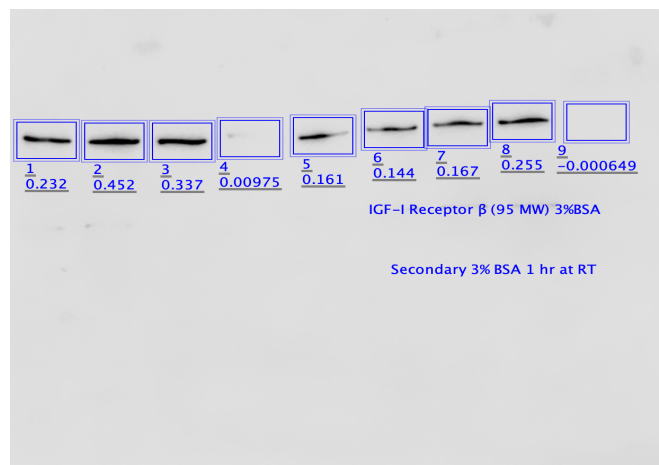

Supplement: Supplementary file 1 — Supplementary Information. [file 41598_2026_35711_MOESM1_ESM.pdf]
